# Evaluation of Germline Structural Variant Calling Methods for Nanopore Sequencing Data

**DOI:** 10.3389/fgene.2021.761791

**Published:** 2021-11-18

**Authors:** Davide Bolognini, Alberto Magi

**Affiliations:** ^1^ Unit of Medical Genetics, Meyer Children’s Hospital, Florence, Italy; ^2^ Department of Information Engineering, University of Florence, Florence, Italy

**Keywords:** bioinformatics, nanopore sequencing, genomics, structural variation, long reads

## Abstract

Structural variants (SVs) are genomic rearrangements that involve at least 50 nucleotides and are known to have a serious impact on human health. While prior short-read sequencing technologies have often proved inadequate for a comprehensive assessment of structural variation, more recent long reads from Oxford Nanopore Technologies have already been proven invaluable for the discovery of large SVs and hold the potential to facilitate the resolution of the full SV spectrum. With many long-read sequencing studies to follow, it is crucial to assess factors affecting current SV calling pipelines for nanopore sequencing data. In this brief research report, we evaluate and compare the performances of five long-read SV callers across four long-read aligners using both real and synthetic nanopore datasets. In particular, we focus on the effects of read alignment, sequencing coverage, and variant allele depth on the detection and genotyping of SVs of different types and size ranges and provide insights into precision and recall of SV callsets generated by integrating the various long-read aligners and SV callers. The computational pipeline we propose is publicly available at https://github.com/davidebolo1993/EViNCe and can be adjusted to further evaluate future nanopore sequencing datasets.

## 1 Introduction

Structural variants (SVs) are defined as DNA rearrangements ≥50 bp and include copy number variants (CNVs; deletions and duplications) as well as insertions, inversions, translocations, and more complex combinations of these described events (Alkan et al., 2011; Sudmant et al., 2015). Although single nucleotide variants (SNVs) were initially thought to contribute the majority of genomic variation in humans (Sachidanandam et al., 2001; Zou et al., 2020), SVs can extend to well over megabases of sequence, accounting for more varying base pairs than any other class of sequence variants (Ho et al., 2020).

Several studies have implicated SVs in human health, with associated phenotypes ranging from cognitive neurological disorders (Rovelet-Lecrux et al., 2006; Pytte et al., 2020) to obesity (Walters et al., 2013) and cancer (Li et al., 2020; Aganezov et al., 2020), among others (Weischenfeldt et al., 2013).

Despite the importance of SVs, they have been largely understudied compared to SNVs because of dominant short-read sequencing technologies hindering their identification, especially in low-complexity regions, which are known to be SV hotspots (Mills et al., 2011). Indeed, it has been shown that from a computational perspective, repeats create ambiguities in short-read alignment and assembly which, in turn, introduces errors in calling genetic variants (Treangen and Salzberg, 2011; Mantere et al., 2019).

Long-read sequencing from Pacific Biosciences and Oxford Nanopore Technologies (ONT) has emerged in recent years (Chaisson et al., 2015; Jain et al., 2016) and proved invaluable in identifying previously intractable DNA sequences (Li and Freudenberg, 2014; Bolognini et al., 2020) and close gaps in the human genome assemblies and unraveling otherwise undetected SVs at population-scale (Beyter et al., 2020; Wu et al., 2021).

The idea of sequencing DNA fragments using a protein nanopore dates back to the 1980s and culminated in the ONT MinION device being released in June 2014 (Deamer et al., 2016). A single MinION flowcell has 512 sensors collecting measurements from 2048 nanopores and currently allows us to sequence a full human genome at 3-4X coverage with read lengths up to ∼800 kbp (Jain et al., 2018). While low-coverage data can be used to detect CNVs at array resolution (Magi et al., 2019), higher throughput facilitates the resolution of the full SV spectrum with base-pair resolution and can be achieved by combining multiple MinION runs (Cretu Stancu et al., 2017) or by sequencing through the high-performance PromethION platform (De Coster et al., 2019).

Thanks to the efforts of the Human Genome Structural Variation (Chaisson et al., 2019) and Genome in a Bottle (GIAB) (Zook et al., 2020) consortia, high-coverage nanopore sequencing data have been released to the research community together with high-quality SV callsets that enable an accurate estimation of precision and recall of SV calling methods. Moreover, with many other studies to follow, such as the All of Us research program and the Human Pangenome project (De Coster et al., 2021), a throughout benchmark of available strategies for the identification and characterization of SVs from nanopore data is greatly needed.

In this article, we present an evaluation of current long-read SV calling pipelines applied to nanopore sequencing data. Specifically, we focus on germline SVs identified by read alignment–based approaches and evaluate each SV caller’s ability to detect genomic breakpoints of different SV types and size ranges and the effects of read alignment, sequencing coverage, variant allele depth, and integration of multiple call sets on SV detection and genotyping. A scalable workflow for SV calling based on the popular workflow language Snakemake (Köster and Rahmann, 2012) is available at https://github.com/davidebolo1993/EViNCe and can be used to reproduce findings described in this article and adapted to future nanopore sequencing datasets.

## 2 Methods

The evaluation workflow used in this work is outlined in short below. Additional details are provided in the accompanying Supplementary Material.

We benchmarked 5 SV calling methods, namely, Sniffles (Sedlazeck et al., 2018), SVIM (Heller and Vingron, 2019), cuteSV (Jiang et al., 2020), npInv (Shao et al., 2018), and pbsv (https://github.com/PacificBiosciences/pbsv), using real ONT PromethION data released by the GIAB consortium for the NA24385 Ashkenazim individual (Shafin et al., 2019) and synthetic ONT data generated using the SV simulator VISOR (Bolognini et al., 2019), aligned to the GRCh37 and GRCh38 versions of the human reference genome, respectively, using the long-read aligners minimap2 (Li, 2018), NGMLR (Sedlazeck et al., 2018), lra (Ren and Chaisson, 2021), and pbmm2 (https://github.com/PacificBiosciences/pbmm2) (Supplementary Datasheet S1).

By randomly down-sampling the original alignments and filtering the generated SV callsets on different numbers of reads supporting a reported SV, we evaluated the influence of various depths of coverage and variant allele depths on SV callers’ ability to detect genomic breakpoints and identify their genotype.

Precision and recall of the SV callsets generated by combining the different long-read aligners and SV callers were calculated using truvari (https://github.com/spiralgenetics/truvari) against the truth SV callsets from GIAB and VISOR. In accordance with similar studies (Gong et al., 2020), the following criteria were used to pick out true-positive calls: 1) the genomic position of the breakpoints identified for a candidate SV must be within a predefined reference distance (500 bp) from at least one SV in the truth callset, 2) the SV type reported for the candidate SV must match the SV type of the SV in the truth callset, and 3) for genotyping, the genotype of the candidate SV must match the genotype of the SV in the truth callset. Candidate SVs absent (and, for genotyping, also those not having a matching genotype) from the truth callset were considered false positives, and *vice versa* for false negatives.

## 3 Results

### 3.1 Nanopore Sequencing Datasets and Truth SV Callsets

For benchmarking, we used the ultra-long ONT data released by the GIAB consortium for the NA24385 individual. These data were generated running 3 flow cells in parallel on the ONT PromethION sequencing platform and yielded ∼157 Gbp throughput. A high-quality callset of insertions and deletions derived from short-, long-, and linked-read sequencing and optical mapping is available for the same individual on the human GRCh37 reference genome and was used as the truth callset. The NA24385 truth SV callset contains 12,745 SVs (with the FILTER “PASS”), divided into 7,281 insertions and 5,464 deletions with the size ranges reported in Supplementary Table S1.

Since not all the SV types are included in the NA24385 truth callset, we additionally generated synthetic ONT data (∼154 Gbp throughput) that we refer to as SI00001 from now on, harboring deletions and insertions as well as inversions, duplications, and translocations using the SV simulator VISOR (average length and standard deviation of reads are ∼15,000 bp and 12,000 bp, respectively, and minimum and maximum identity of sequences is set to ∼88% and ∼98%). In greater detail, we inserted 10,676, randomly generated, heterozygous SVs in chromosomes 1 to 22, X and Y of the human GRCh38 reference genome, divided into 5,027 deletions, 5,027 insertions, 300 duplications, 300 inversions, and 22 cut-paste translocations (Supplementary Table S1).

Further references to the data mentioned above are available in the Data Availability Statement section.

### 3.2 Long-Read Aligners and SV Callers

NA24385 and SI00001 ONT reads were aligned to the GRCh37 and GRCh38 decoy versions of the human reference genome, respectively, using the long-read aligners minimap2, NGMLR, lra, and pbmm2 (Supplementary Table S2). Read depth of the resulting alignments was calculated using mosdepth (Pedersen and Quinlan, 2017) and additional alignment statistics using NanoPack (De Coster et al., 2018).

As shown in Supplementary Figure S1, minimap2 produced the highest coverage alignments (∼138 Gbp aligned in the NA24385 dataset and ∼142 Gbp in the SI00001 dataset) and lra the lowest (∼116 Gbp aligned in the NA24385 dataset and ∼135 Gbp in the SI00001 dataset), with NGMLR and pbmm2 performing intermediately (∼127 Gbp and ∼128 Gbp aligned in the NA24385 dataset; ∼137 Gbp and ∼139 Gbp aligned in the SI00001 dataset). Alignments from NGMLR and pbmm2 hit the highest N50 score (∼63 Kbp for the NA24385 dataset and ∼22 Kbp for the SI00001 dataset), minimap2 the lowest (∼49 Kbp for the NA24385 dataset and ∼21 Kbp for the SI00001 dataset), and lra placed in-between (∼55 Kbp for the NA24385 dataset and ∼21 kbp for the SI00001 dataset). Among the tested aligners, minimap2 was the fastest (i.e., ∼26 min to align 100,000 reads, randomly sampled from the NA24385 dataset, using a single core on our SUSE Linux Enterprise Server—average of three consecutive measurements) and NGMLR the slowest (∼190 min, ∼7 times slower than minimap2), with lra and pbmm2 performing similarly (∼30 and ∼34 min, respectively).

We used the generated NA24385 and SI00001 alignments to benchmark 5 SV calling methods, namely, Sniffles, SVIM, cuteSV, npInv, and pbsv. We tuned the settings of the different SV callers to report only variants ≥50 bp, having at least 2 reads supporting the identified SVs (Supplementary Table S2).

While Sniffles, SVIM, cuteSV, and pbsv can detect all SV types, npInv is developed specifically to identify inversions. Because the recommended aligner for pbsv is pbmm2, neither was pbmm2 tested with other SV callers nor was pbsv with other long-read aligners. Furthermore, because pbmm2 wraps minimap2 but uses lower gap penalties for SV discovery, results from pbsv are reported after minimap2 alignment in all figures and tables of this article.

Supplementary Table S3 summarizes by SV type the SVs identified by the different combinations of long-read aligners and SV callers in the NA24385 and SI00001 datasets, before and after filtering for high-quality SVs (i.e., SVs with the FILTER “PASS” that fall in assembled chromosomes only and are supported by ≥ 10 reads). The size distribution of the high-quality SVs is shown in Supplementary Figure S2 (NA24385) and Supplementary Figure S3 (SI00001). SVIM following minimap2 alignment detected more deletions (9,566) and insertions (12,818) than the other aligner–SV caller combinations in the NA24385 dataset, pbsv more duplications (1941), and cuteSV more inversions (156) and translocation breakpoints (37), following NGMLR and minimap2 alignment, respectively. For the SI00001 dataset, cuteSV following minimap2 alignment detected more deletions (4,763) and insertions (4,320), SVIM after minimap2 more duplications (358), cuteSV after NGMLR more inversions (590), and pbsv more translocation breakpoints (i.e., BNDs, 39).

For each aligner, we also calculated the number of SVs overlapping between the high-quality SV callsets and the corresponding truth callset using SURIVOR (Jeffares et al., 2017). SVs were considered to be shared among the callsets if their distance was ≤500 bp, as measured pairwise between breakpoints, and their type was concordant. As shown in the resulting upset plots for minimap2 (Supplementary Figure S4), the largest overlap is shared between the truth callset and the different SV callers (with the obvious exception of npInv) and mostly contains deletions (4,022 for the NA24385 dataset and 3,368 for SI00001) and insertions (4,054 for the NA24385 dataset and 3,101 for SI00001). Comparable results are obtained with the NGMLR (Supplementary Figure S5) and lra (Supplementary Figure S6) alignments.

### 3.3 Structural Variant Caller’s Performances

**FIGURE 1:**
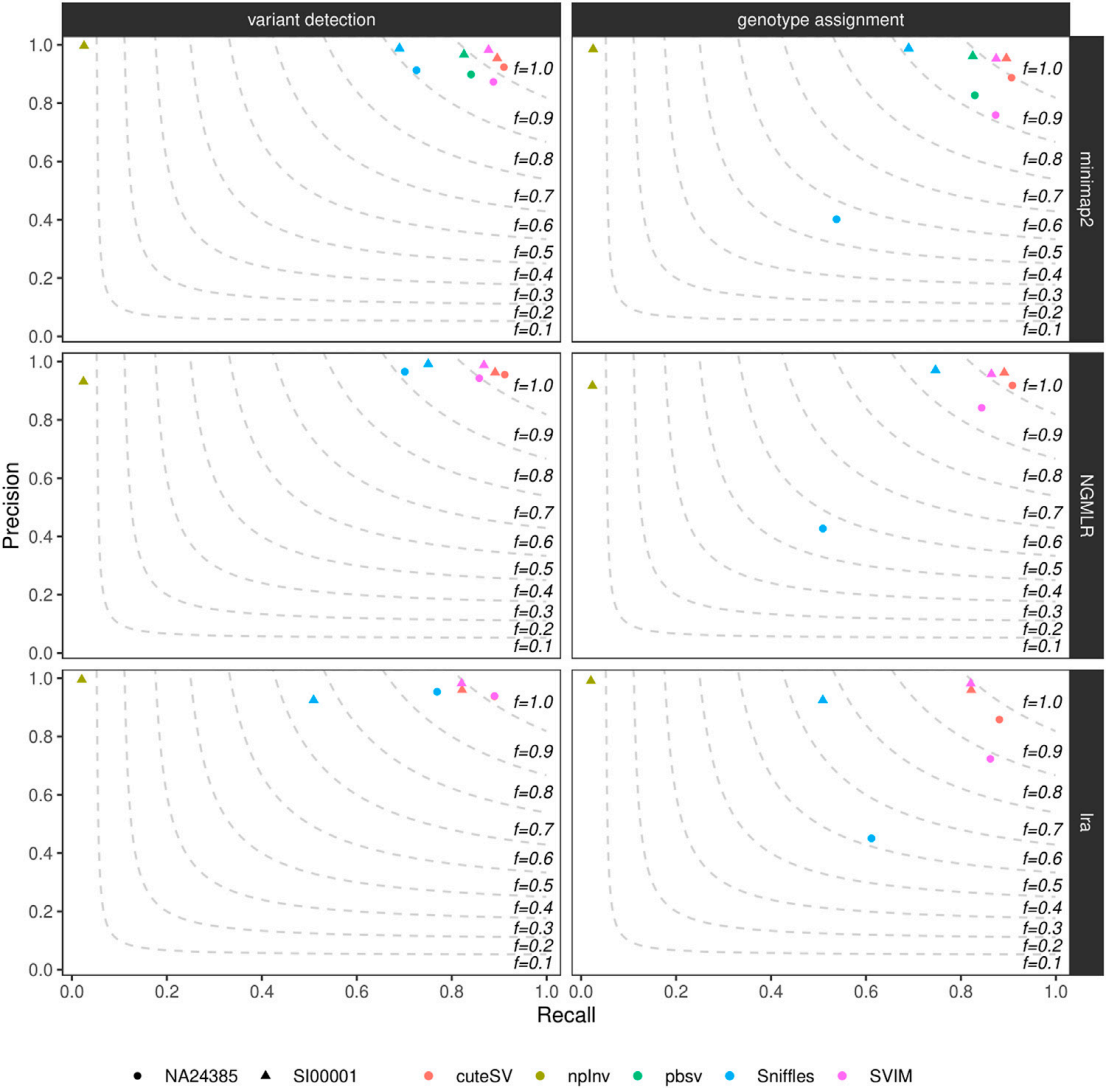
Precision (y axis), recall (x axis) and F-score (dashed lines) of the high-quality SV callsets from Sniffles, SVIM, cuteSV, npInv and pbsv (hue palette) after minimap2 (top panels), NGMLR (mid panels) and lra (bottom panels) alignments. Results for both SV calling (left panels) and genotyping (right panels) in the NA24385 (circle symbol) and SI00001 (triangle symbol) datasets are shown.

We calculated precision, recall, and F-score (i.e., the harmonic mean of precision and recall) of the generated high-quality SV callsets using truvari (see Methods and Supplementary Datasheet S2). Figure 1 and Supplementary Table S4 show these findings for the datasets tested. CuteSV following NGMLR alignment reached the highest F-score at SV calling and genotyping (∼0.93 and ∼0.91, respectively) in the NA24385 dataset; for the SI00001 dataset, SVIM after minimap2 reached the highest F-score at SV calling (∼0.93) while cuteSV after NGMLR reached the highest F-score at SV genotyping (∼0.92). Overall, cuteSV, SVIM, and pbsv performed similarly well at SV calling, with F-score values ∼0.90. With the obvious exception of npInv, which is specifically tailored to identify inversions, Sniffles achieved the lowest recall, especially in the SI00001 dataset after lra alignment.

Supplementary Figure S7 illustrates precision and recall of the high-quality SV callsets when resolved by SV type. CuteSV, SVIM, and pbsv compared favorably to Sniffles for the detection of deletions in both NA24385 and SI00001 datasets (F-score > 0.90 vs. F-score < 0.90), while for insertions, only cuteSV, after NGMLR in the NA24385 dataset and after NGMLR/minimap2 in SI0001, hit F-score > 0.90. With respect to duplications, SVIM and cuteSV following NGMLR alignment outperformed the other combinations, and for inversions, SVIM after minmimap2, Sniffles after minimap2 and NGMLR, npInv after minimap2, and pbsv reached F-score > 0.90. Last, pbsv and SVIM following minimap2 alignment had the highest F-score for the detection of translocations (F ∼0.90). Notably, no high-quality duplications or translocations were reported by any SV callers when tested on alignments from lra.

The number of true-positive, false-positive, and false-negative SV calls relative to their length is reported in Supplementary Figure S8. The peaks in the NA24385 dataset at ∼300 bp and ∼6,000 bp correspond to SVs involving Alu and L1 elements, respectively (Audano et al., 2019), while those in the SI00001 dataset at ∼1,000 bp and ∼10,000 bp correspond to the average size of simulated SVs.

**FIGURE 2:**
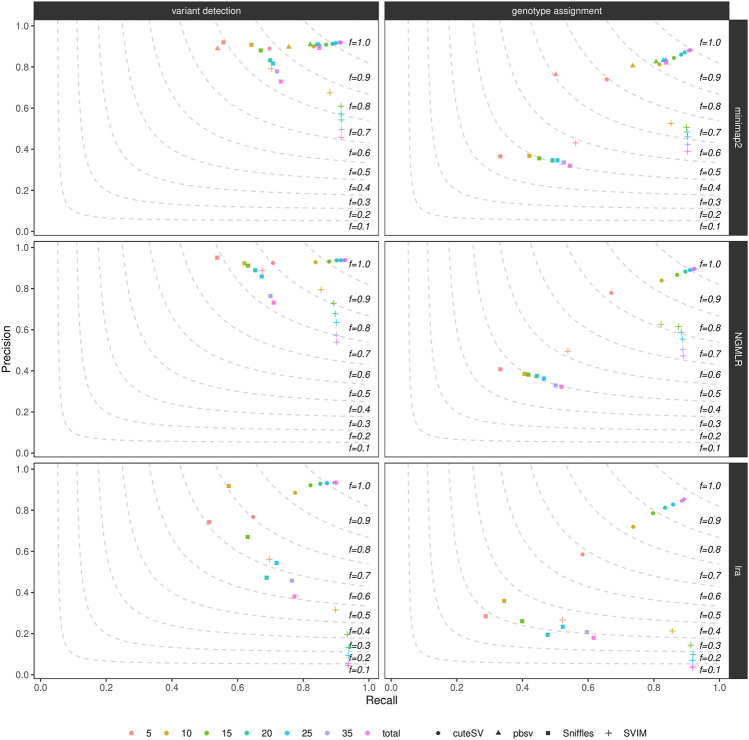
Precision (y axis), recall (x axis) and F-score (dashed lines) of the SV callers Sniffles (square symbol), SVIM (cross symbol), cuteSV (circle symbol) and pbsv (triangle symbol) after minimap2 (top panels), NGMLR (mid panels) and lra (bottom panels) alignments. Results for both SV calling (left panels) and genotyping (right panels) are reported. The plot shows the influence of average genome coverage after down-sampling NA24385 alignments to different fractions (5X, 10X, 15X, 20X, 25X, 35X–hue palette) of the original coverage (total) on SV callers' performances.

By randomly down-sampling the NA24385 and SI00001 original alignments to various fractions of the original datasets (i.e., 5X, 10X, 15X, 20X, 25X, and 35X), we evaluated the influence of genome coverage on SV callers’ precision and recall. Figure 2, Supplementary Figure S9, and Supplementary Table S5 show these findings for the NA24385 and SI00001 datasets, respectively. While recall for both SV calling and genotyping increased significantly when moving on from low- (i.e., 5X) to mid-range (i.e., 15X-20X) coverage, this effect was less marked for higher depths of coverage and came at the cost of a reduction in precision for Sniffles (NA24385 dataset) and SVIM (NA24385 and SI00001 datasets). For low-coverage NA24385 data, CuteSV after NGMLR alignment hit the highest F-score (∼0.80 for SV calling and ∼0.72 for genotyping) and Sniffles after lra the lowest (∼0.60 for SV calling and ∼0.28 for SV genotyping). For low-coverage SI00001 data, cuteSV after NGMLR alignment hit the highest F-score at SV calling (∼0.70) and pbsv the lowest (∼0.43), while Sniffles after NGMLR reached the highest F-score at SV genotyping (∼ 0.61) and SVIM after lra the lowest (∼ 0.32).

**FIGURE 3:**
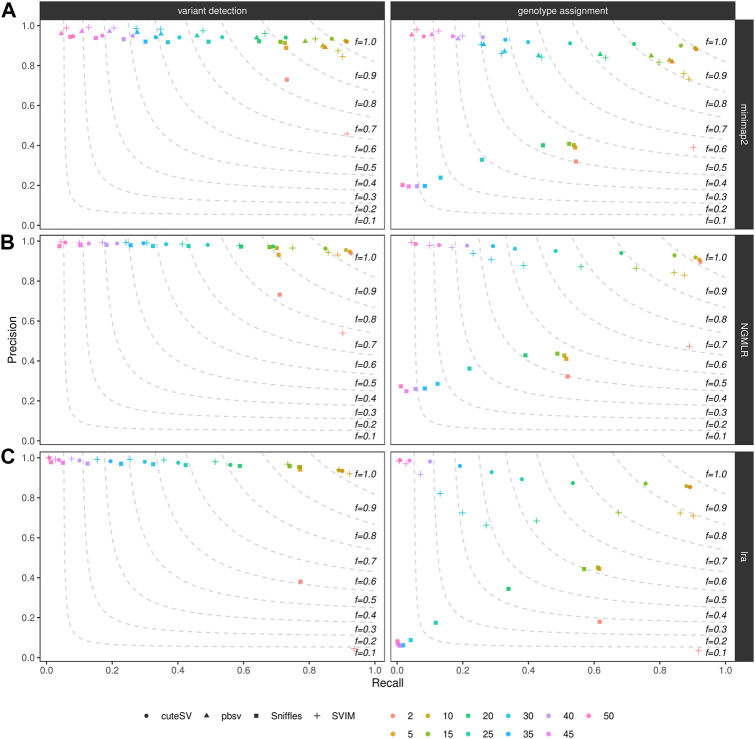
Precision (y axis), recall (x axis) and F-score (dashed lines) of the SV callers Sniffles (square symbol), SVIM (cross symbol), cuteSV (circle symbol) and pbsv (triangle symbol) after minimap2 (top panels), NGMLR (mid panels) and lra (bottom panels) alignments. Results for both SV calling (left panels) and genotyping (right panels) are reported. The plot shows the influence of the number of reads minimally supporting a SV (2, 5, 10, 15, 20, 25, 30, 35, 40, 45, 50–hue palette) on SV callers' performances for the NA24385 dataset.

We furthermore examined how filtering on the minimum number of reads supporting the variant alleles affects precision and recall of SV callers. As expected, for all the combinations tested in the NA24385 (Figure 3) and SI00001 (Supplementary Figure S10) datasets (see also Supplementary Table S6), the recall was the highest (and precision the lowest) when less support for a candidate SV (i.e., 2) is used and decreased (while precision increased) when higher support was required (i.e., up to 50 supporting reads for the NA24385 dataset and up to 25 for SI00001). From our tests, a good trade-off between precision and recall was achieved when SVs were minimally supported by 5–10 reads (see above for details on the F-score when filtering on a minimum number of 10 supporting reads).

**FIGURE 4:**
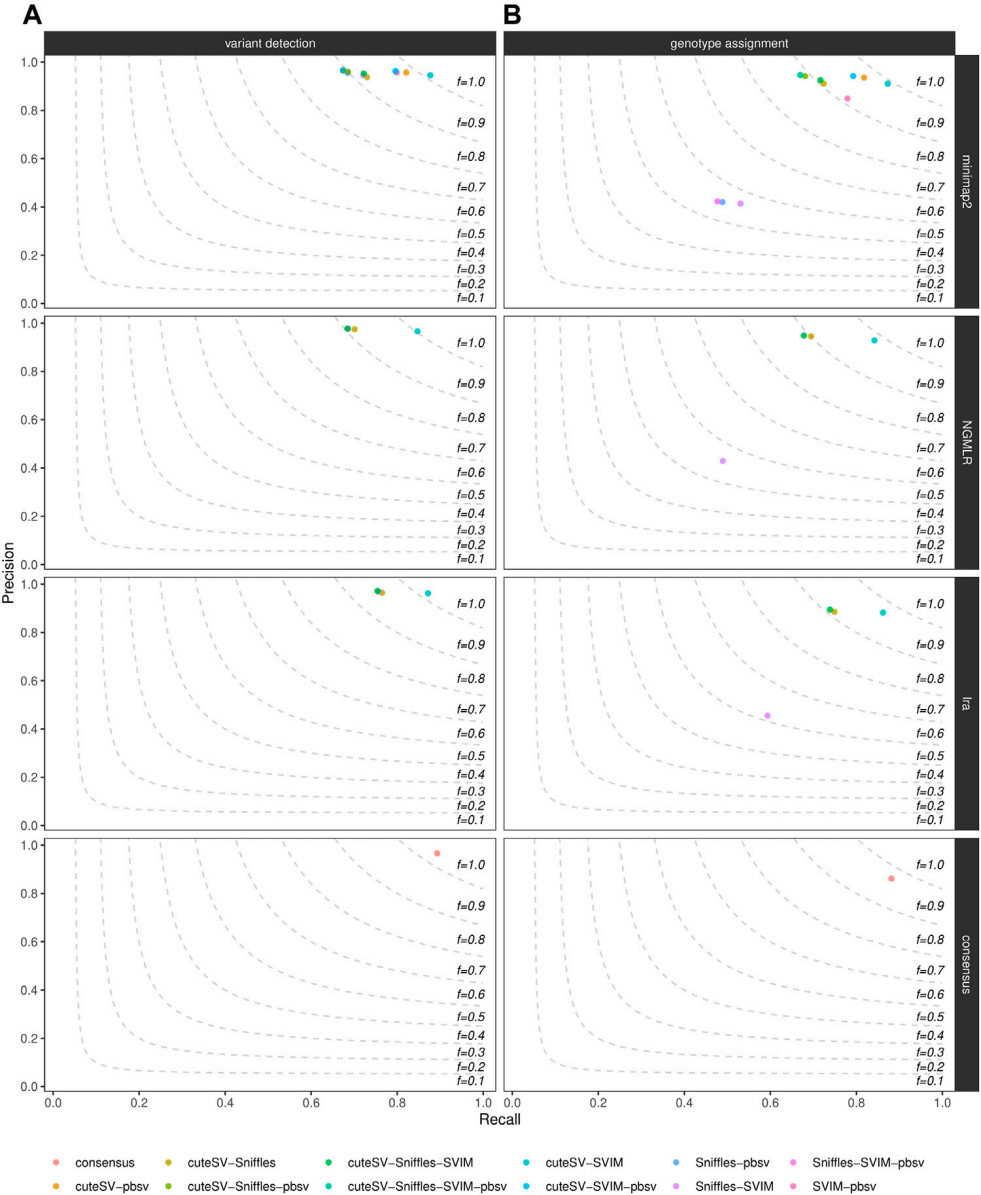
Precision (y axis), recall (x axis) and F-score (dashed lines) of the combination of the SV callers Sniffles, SVIM, cuteSV and pbsv (hue palette) after minimap2, NGMLR and lra alignments as well as after consensus generation (top-to-bottom panels). Results for both SV calling (left panels) and genotyping (right panels) are reported. The plot shows the influence of the integration of multiple high-quality callsets on reducing false positive calls in the NA24385 dataset.

Last, we evaluated how much of the false positive rate from individual SV callsets we could reduce by integrating multiple SV callers for the same sample. For each aligner tested, we calculated precision and recall of all the combinations of the SV callers tested, as shown in Figure 4 for the NA24385 dataset and Supplementary Figure S11 for the SI00001 dataset. An additional consensus callset including SVs supported by at least 4 (minimap2) or 3 (NGMLR and lra) SV callers and at least 2 long-read aligners was also produced. Results are documented in Supplementary Table S7 as well. The different combinations of SV callsets were generated using SURVIVOR, following the strategy described in the previous section. For the NA24385 dataset, combining high-quality SV calls from cuteSV, Sniffles, and SVIM led to a ∼2% increase in precision at both SV calling and ∼3% at SV genotyping with respect to the corresponding highest precision values reached by Sniffles (∼0.96) and cuteSV (∼0.92), respectively, after NGMLR alignment. For SV calling, the consensus callset reached comparable precision (∼0.96) but improves on recall (∼0.89) with respect to the other combinations tested. For the SI0001 dataset, combining multiple SV callsets did not show significant improvement over precision of single SV callsets. For instance, most of the combinations tested hit ∼1.00 precision at SV calling, but Sniffles alone after NGMLR reached precision > 0.99.

## 4 Discussion

While short-read sequencing has been considered the gold standard for the majority of sequencing projects for years (Roberts et al., 2021), such data have biases in whole-genome sequencing studies due to the uneven coverage of regions with high/low GC and difficulty of mapping short reads in low-complexity regions. Long-read sequencing has already proved invaluable in overcoming these limitations, improving on short reads for the resolution of SVs in comparative and clinical studies (Sanchis-Juan et al., 2018).

In this article, we provided a succinct yet comprehensive evaluation of long-read SV calling pipelines applied to ONT data. In particular, we focused on germline SVs, and as such, our findings are likely not reproducible in different contexts, such as somatic variant calling, for which alternative strategies exist (Shiraishi et al., 2020).

We tested four general-purpose SV callers (Sniffles, SVIM, cuteSV, and pbsv) and a tool tailored specifically to inversions (npInv) across four long-read aligners (minimap2, NGMLR, lra, and pbmm2) using both real and simulated ONT data. In particular, we used the ultra-long ONT reads released by the GIAB consortium for the NA24385 Ashkenazim individual, for which a truth set of deletions and insertions based on the integration of multiple technologies is available, and synthetic long reads generated using the SV simulator VISOR (SI00001) to complement SVs missing in the real dataset (inversions, duplications, and translocations). Also, although the NA24385 truth set from GIAB is assumed to be sufficiently complete, which is supported by the fact that the majority of the SVs identified by the different SV calling pipelines is shared with the ground truth, a consistent number of deletions and insertions identified by multiple SV callers are absent from the truth callset, suggesting that at least part of them could have been missed in the ground truth.

We first calculated the precision, recall, and F-score of the different SV calling pipelines after filtering for high-quality variants (“PASS” SVs not falling in decoy contigs and supported by at least 10 reads—which is the default for SV callers like Sniffles and cuteSV) and evaluated the impact of each SV type and various SV sizes on the SV callers’ performances. In accordance with prior evaluations (De Coster et al., 2019; Zhou et al., 2019), we observed the highest precision at SV calling with Sniffles following NGMLR alignment in both the NA24385 (∼0.96) and SI00001 (∼0.99) datasets but at a cost of low recall, with most of the false-negative SVs being shorter than 500 bp in the real dataset. However, cuteSV, SVIM, and pbsv all performed better than Sniffles in terms of the F-score across the different aligners, and Sniffles also hit the lowest F-score values at SV genotyping in both datasets. When taking into account the individual SV types, cuteSV, SVIM, and pbsv had the best performances for the detection of deletions, and cuteSV after NGMLR (NA24385 and SI0001) and minimap2 (SI00001) hit the highest F-score for the detection of insertions and duplications together with SVIM after NGMLR. For inversions, SVIM (after minimap2), Sniffles, npInv (after minimap2 or NGMLR), and pbsv all hit an F-score of ∼ 0.9 or higher, while SVIM after minimap2 and pbsv performed better than the other aligner–SV caller combinations for the detection of translocation breakpoints. Notably, none of the SV callers tested were able to identify high-quality duplications or translocations after lra alignment, especially in the SI00001 dataset where they are known to occur. Manual investigation of the variant files generated by the different SV callers before filtering revealed that most duplications and translocations had few supporting reads ( < 3 in most cases) and were not flagged as “PASS.” As a consequence, being more permissive with the filters used could improve on the detection of these SV categories in datasets aligned with lra.

When further evaluating the influence of genome coverage on the SV caller’s performances, we concluded that adding more than 15X–20X produced only little increment in sensitivity but was associated with a marked decrease in precision for Sniffles and SVIM. On the other hand, a slight increase in precision could be reached by combining multiple callsets and the consensus from cuteSV, Sniffles, and SVIM after NGMLR hit the highest precision values in our tests. Last, we highlighted that having at least 5–10 reads supporting a called SV represents a good trade-off to optimize precision and recall.

Given the results presented in this article, we recommend using cuteSV for the initial assessment of the data as it achieves substantial precision and recall at both SV calling and genotyping, even when analyzing low-coverage data. Because the choice of the pipeline could depend on the need of the users to retrieve SV calls with either high precision or high recall, we conclude that Sniffles should be preferred when looking for high precision, while cuteSV or SVIM should be preferred when high recall is required. However, due to low F-score values at SV genotyping, we do not recommend using Sniffles for an accurate estimation of the zygosity of SV calls, as it often misclassified or missed heterozygous variants in our tests. Combining SV calls from cuteSV, Sniffles, and SVIM can be helpful in further reducing the final false positive rate.

## Data Availability

The datasets presented in this study can be found in online repositories. The names of the repository/repositories and accession number(s) can be found in the article/Supplementary Material.
