# Supplementary material for: Evaluation of Germline Structural Variant Calling Methods for Nanopore Sequencing Data
Source: Front Genet. 2021 Nov 18;12:761791. doi: 10.3389/fgene.2021.761791 (PMC8637281; doi:10.3389/fgene.2021.761791)
Supplement: Supplementary file 1 [file DataSheet1.PDF]

Supplementary Information

Evaluation of germline structural variant calling methods for nanopore sequencing data

Davide Bolognini* and Alberto Magi

Supplementary Notes

Supplementary Note S1

Originally, we assessed more long-read aligners and SV callers than those eventually tested in this paper.
In addition to minimap2 [6], NGMLR [9], lra [8] and pbmm2 (`https://github.com/PacificBiosciences/pbmm2`), we included the long-read aligners GraphMap [11] and LAST [5] in our preliminary tests performed on the NA24385 ultra-long nanopore dataset (see the Method and Result sections). We excluded GraphMap because it was the most resource-intensive aligner and crashed multiple times on our SUSE Linux Enterprise Server due to extremely high memory-consumption peaks, as also reported by others [7]. Among the others, LAST was by far the slowest (∼3-4 times slower than NGMLR, see Result), precluding its inclusion in the design of this analysis pipeline (and, in principle, most of the others).
Further to Sniffles [9], SVIM [3], cuteSV [4], npInv [10] and pbsv (`https://github.com/PacificBiosciences/pbsv`), we included the SV callers NanoSV [1] and Picky [2] in the initial evaluation on the NA24385 dataset. Due to the cumbersome running times (one possible strategy to reduce the user waiting-time is to run NanoSV in parallel on each chromosome but this prevents the detection of inter-chromosomal alterations, that is translocations) and the absence of active support from the original authors (the package `https://github.com/mroosmalen/nanosv` is probably unmaintained) we excluded NanoSV. Because the authors suggest to use Picky in combination with alignments from LAST, for the reasons explained above, we decided not to include this tool in the final evaluation as well.

Supplementary Note S2

We used truvari (`https://github.com/spiralgenetics/truvari`) to calculate the number of true positive (TP), false positive (FP) and false negative (FN) calls and derived precision (P), recall (R) and F-score (F) of the assessed SV callers (see Method).
For SV calling, given a truth callset (A) and a comparison callset (B), TP_A identifies the number of matching calls from A, TP_B the number of matching calls from B, FP the number of non-matching calls from B and FN the number non-matching calls from A. P_{call}, R_{call} and F_{call} were then calculated as follows:

$$P_{call} = \frac{TP_B}{TP_B + FP}$$

$$R_{call} = \frac{TP_A}{TP_A + FN}$$

$$F_{call} = 2 * \frac{P_{call} * R_{call}}{P_{call} + R_{call}}$$

For SV genotyping, TP_{AM} identifies TP calls from A with genotype match, TP_{AN} TP calls from A without genotype match, TP_{BM} TP calls from B with genotype match and TP_{BN} TP calls from B without genotype match. P_{geno}, R_{geno} and F_{geno} were then calculated as follows:

$$P_{geno} = \frac{TP_{AM}}{TP_{AM} + FP + TP_{AN}}$$

$$R_{geno} = \frac{TP_{BM}}{\frac{TP_{BM}}{FN}}$$

$$F_{geno} = 2 * \frac{P_{geno} * R_{geno}}{P_{geno} + R_{geno}}$$

Supplementary Figures

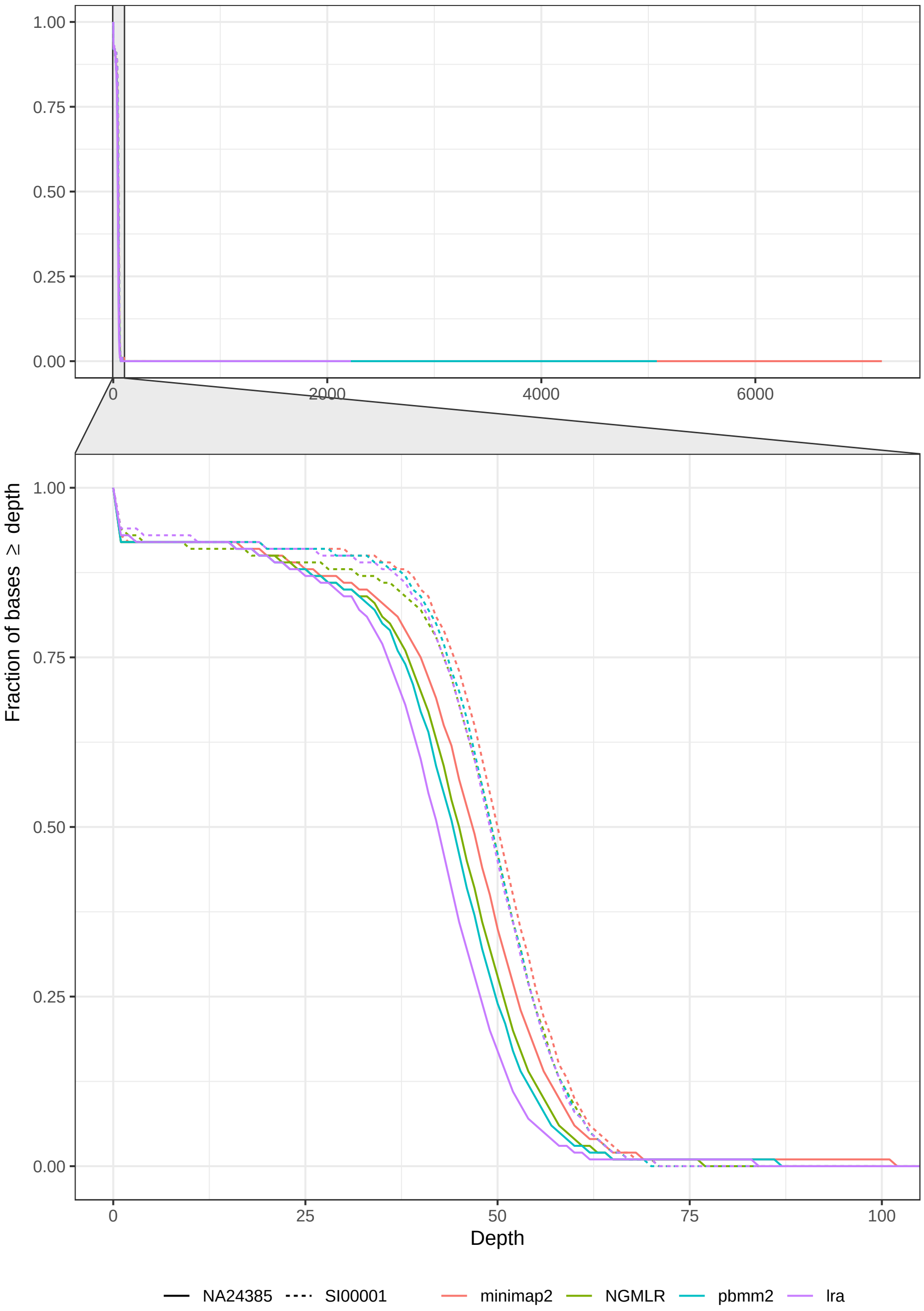

Figure S1: Read depth calculated using mosdepth for the NA24385 (solid line) and SI00001 (dashed line) datasets after alignment with mimimap2, NGMLR, pbmm2 and lra (hue palette). The plot shows the proportion of the reference genome (y axis) covered at least by a certain number of reads (x axis).

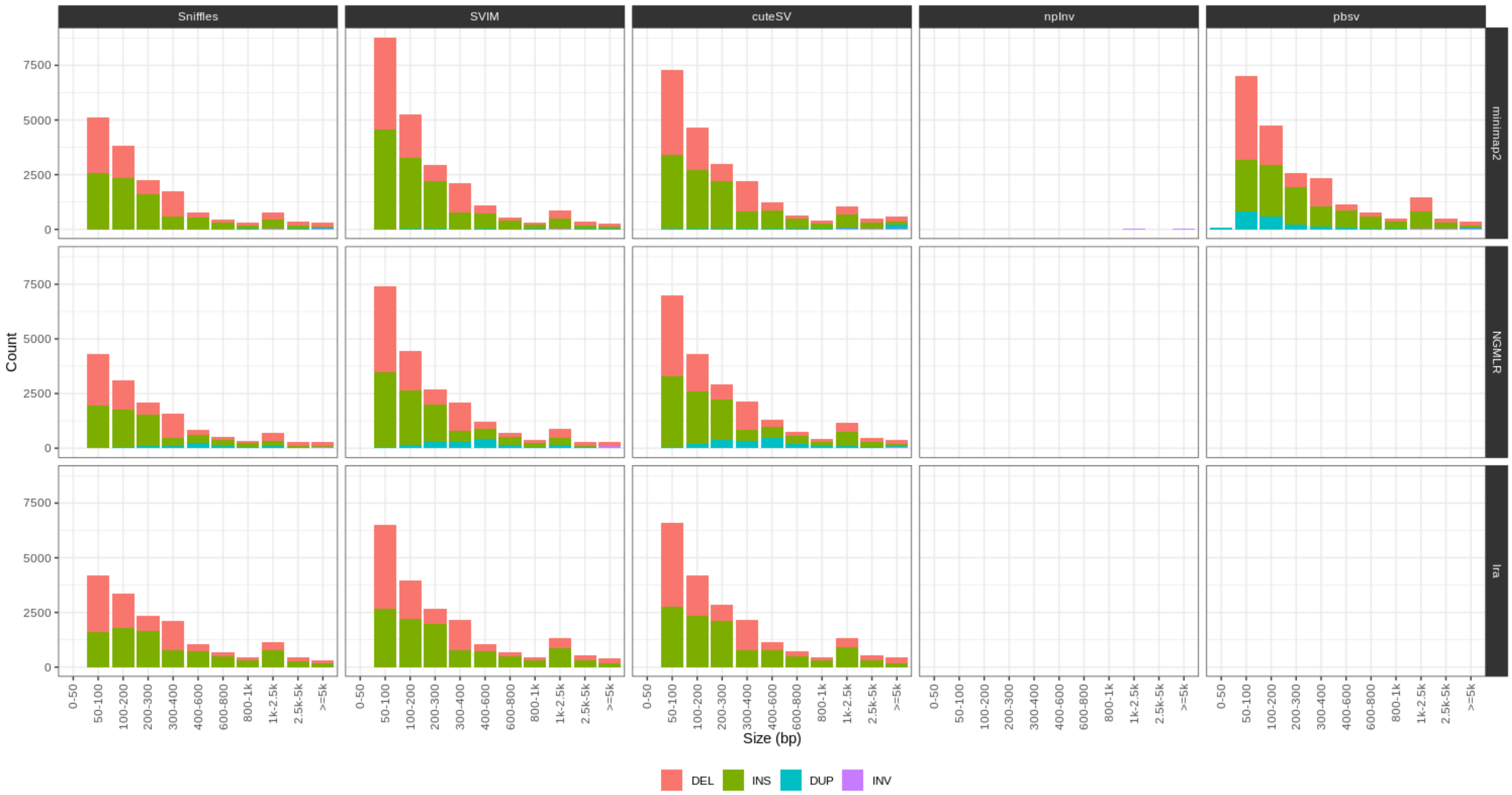

Figure S2: Stacked barchart with counts (y axis) of deletions, insertions, duplications and inversions (hue palette) for each SV size (x axis) in the NA24385 dataset, as reported by truvari for the different SV callers (Sniffles, SVIM, cuteSV, npInv and pbsv, left-to-right panels) across the different aligners (minimap2, NGMLR, lra, top-to-bottom panels).

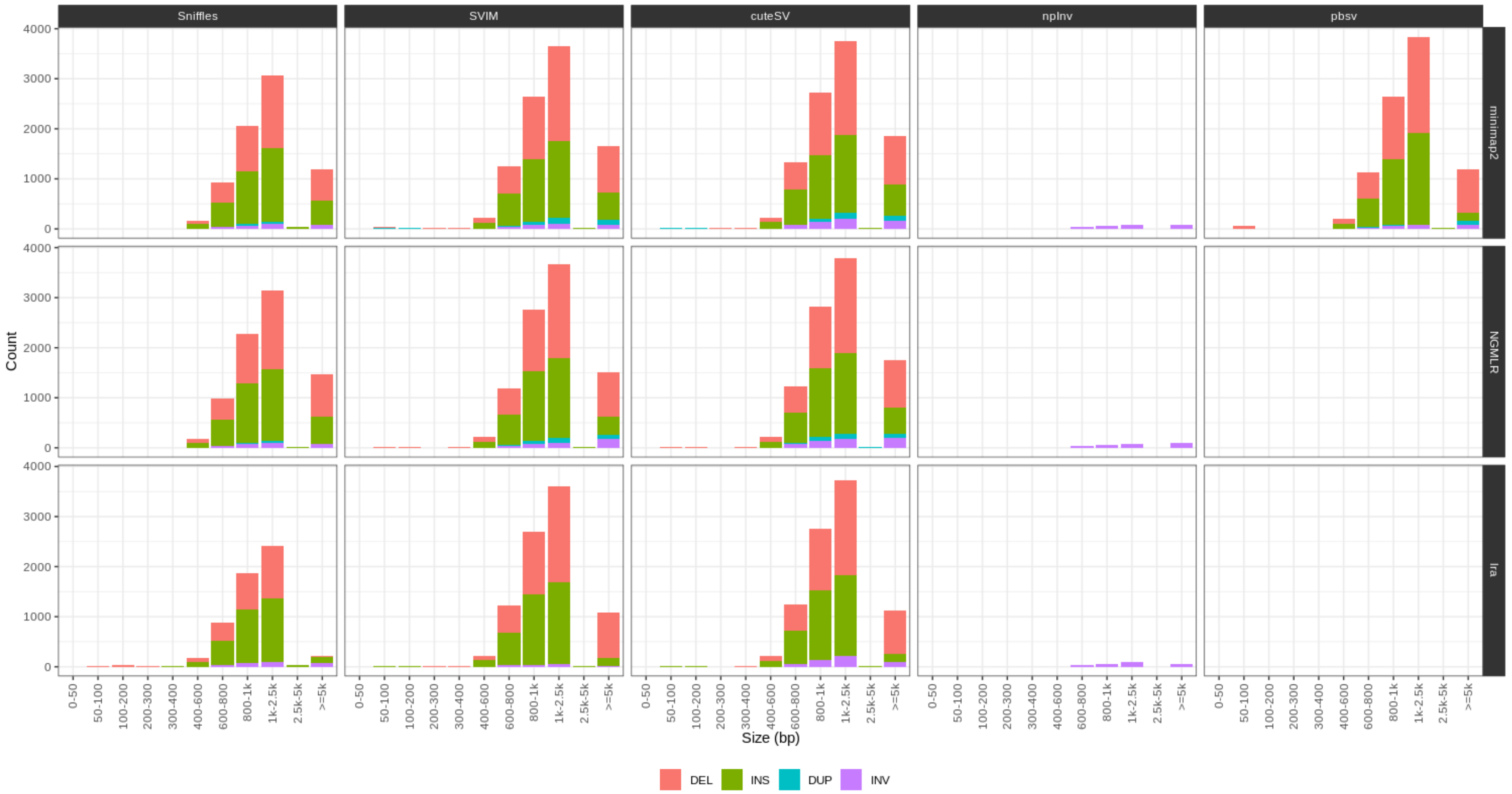

Figure S3: Stacked barchart with counts (y axis) of deletions, insertions, duplications and inversions (hue palette) for each SV size (x axis) in the SI00001 dataset, as reported by truvari for the different SV callers (Sniffles, SVIM, cuteSV, npInv and pbsv, left-to-right panels) across the different aligners (minimap2, NGMLR, lra, top-to-bottom panels).

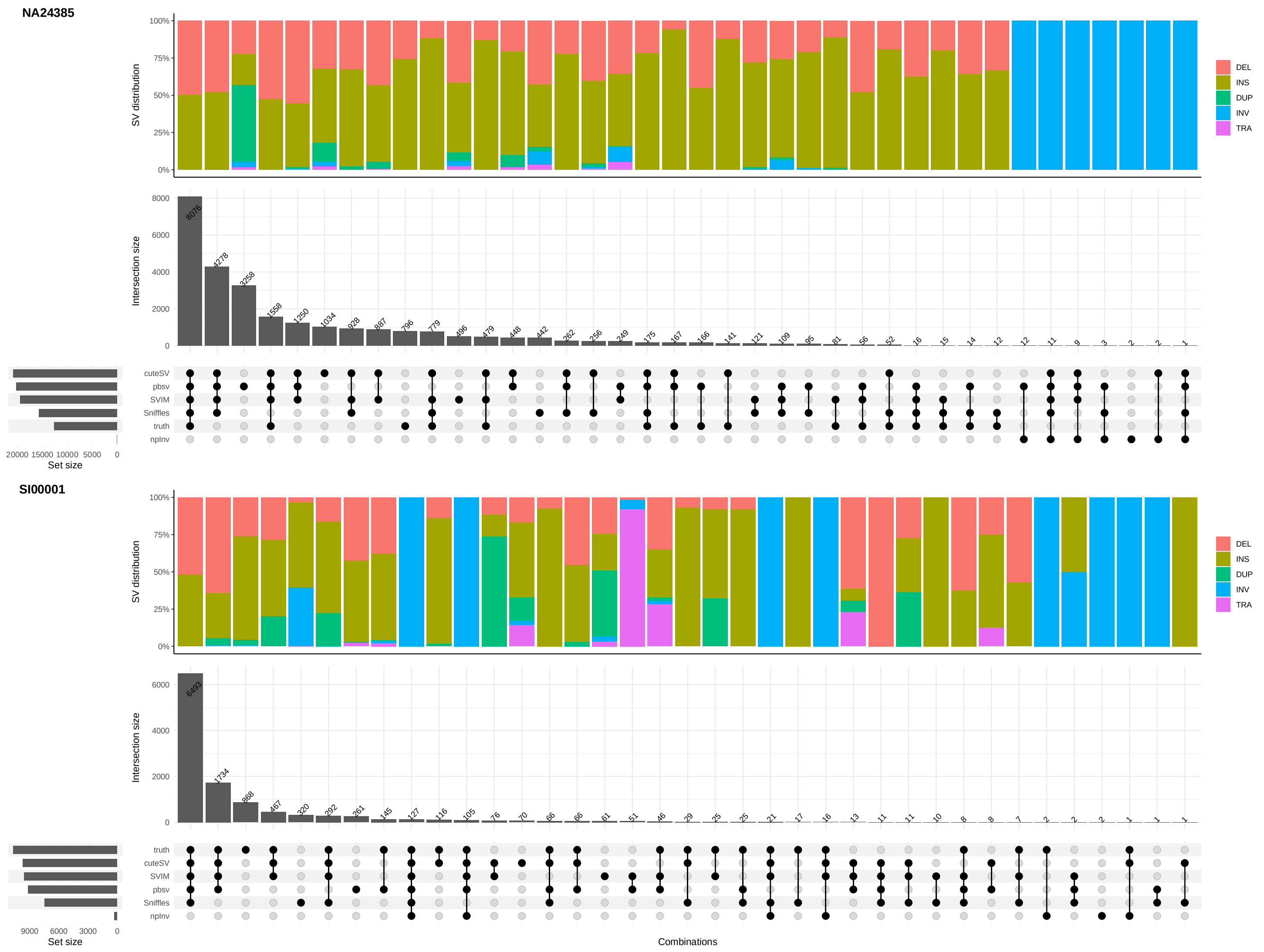

Figure S4: Upset plot of the SVs (hue palette) identified by the different SV callers (Sniffles, SVIM, cuteSV, npInv and pbsv) after minimap2 alignment for the NA24285 (upper panel) and SI00001 (lower panel) datasets. The height of the vertical bars indicates the number of overlapping SVs across the SV callers marked by the grey dots and connecting lines below. The height of the horizontal bars indicates the total number of SVs per SV callset. The companion stacked barchart indicates the SV composition of each intersection.

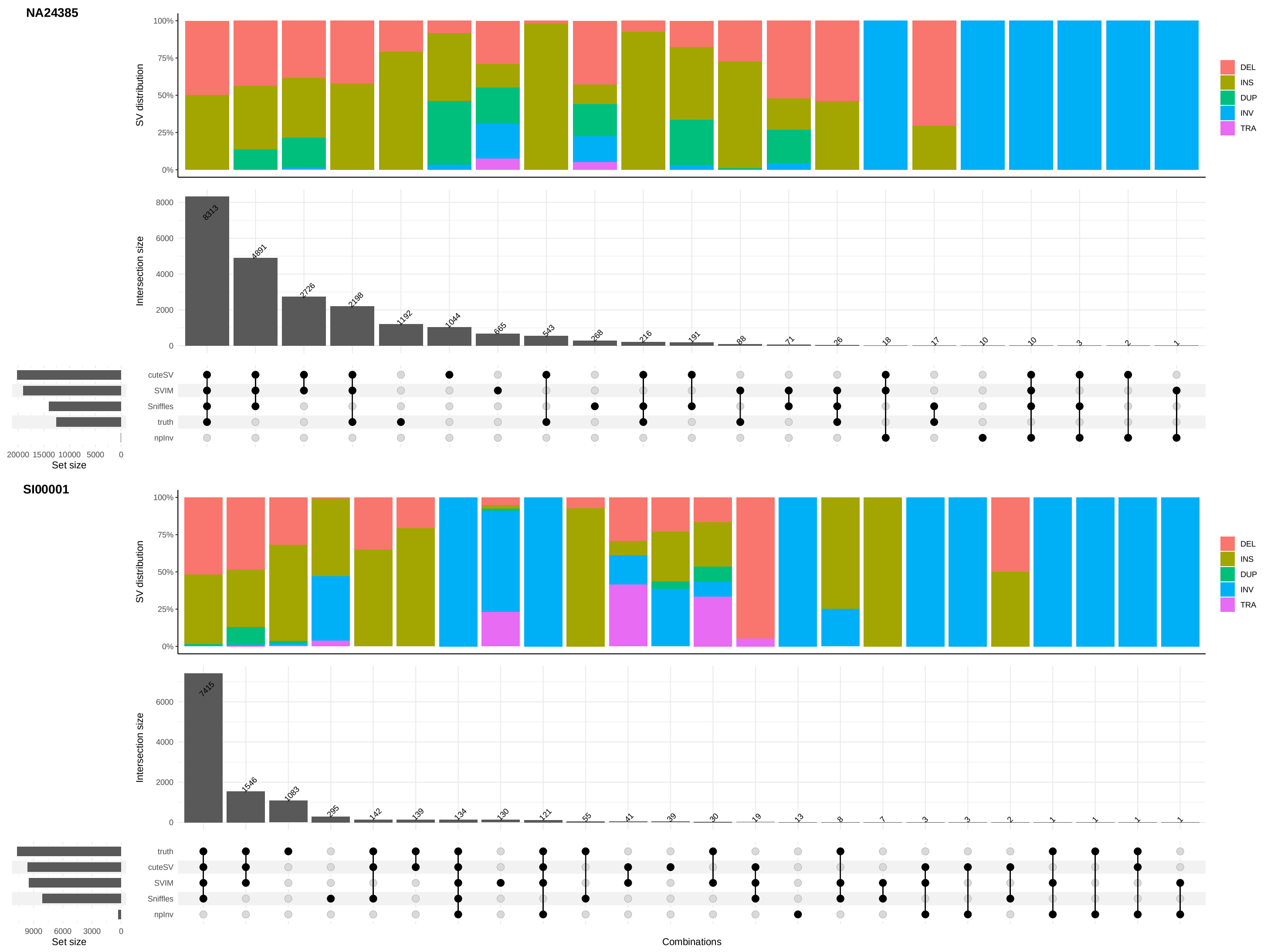

Figure S5: Upset plot of the SVs (hue palette) identified by the different SV callers (Sniffles, SVIM, cuteSV, npInv and pbsv) after NGMLR alignment for the NA24285 (upper panel) and SI00001 (lower panel) datasets. The height of the vertical bars indicates the number of overlapping SVs across the SV callers marked by the grey dots and connecting lines below. The height of the horizontal bars indicates the total number of SVs per SV callset. The companion stacked barchart indicates the SV composition of each intersection.

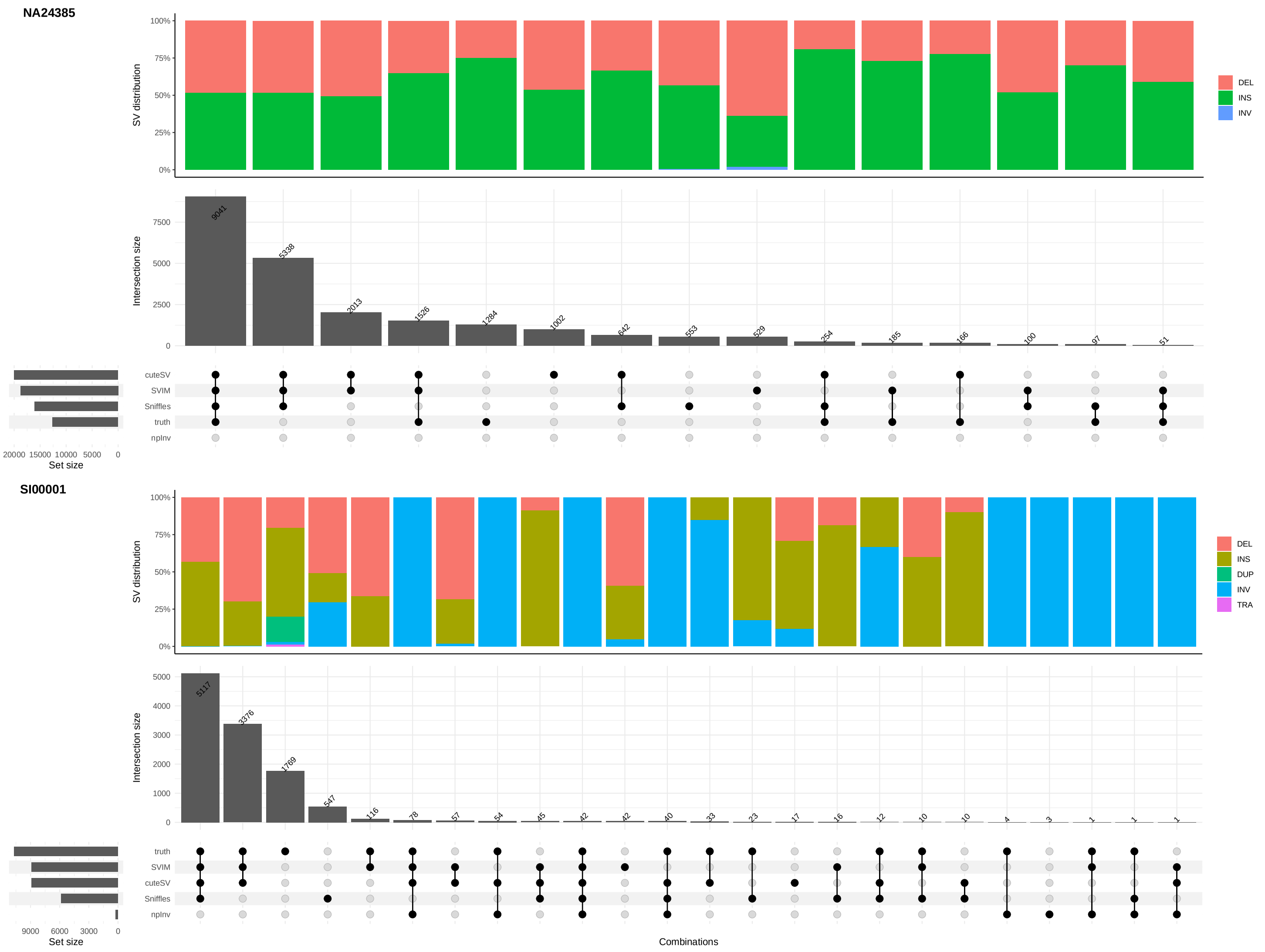

Figure S6: Upset plot of the SVs (hue palette) identified by the different SV callers (Sniffles, SVIM, cuteSV, npInv and pbsv) after lra alignment for the NA24285 (upper panel) and SI00001 (lower panel) datasets. The height of the vertical bars indicates the number of overlapping SVs across the SV callers marked by the grey dots and connecting lines below. The height of the horizontal bars indicates the total number of SVs per SV callset. The companion stacked barchart indicates the SV composition of each intersection.

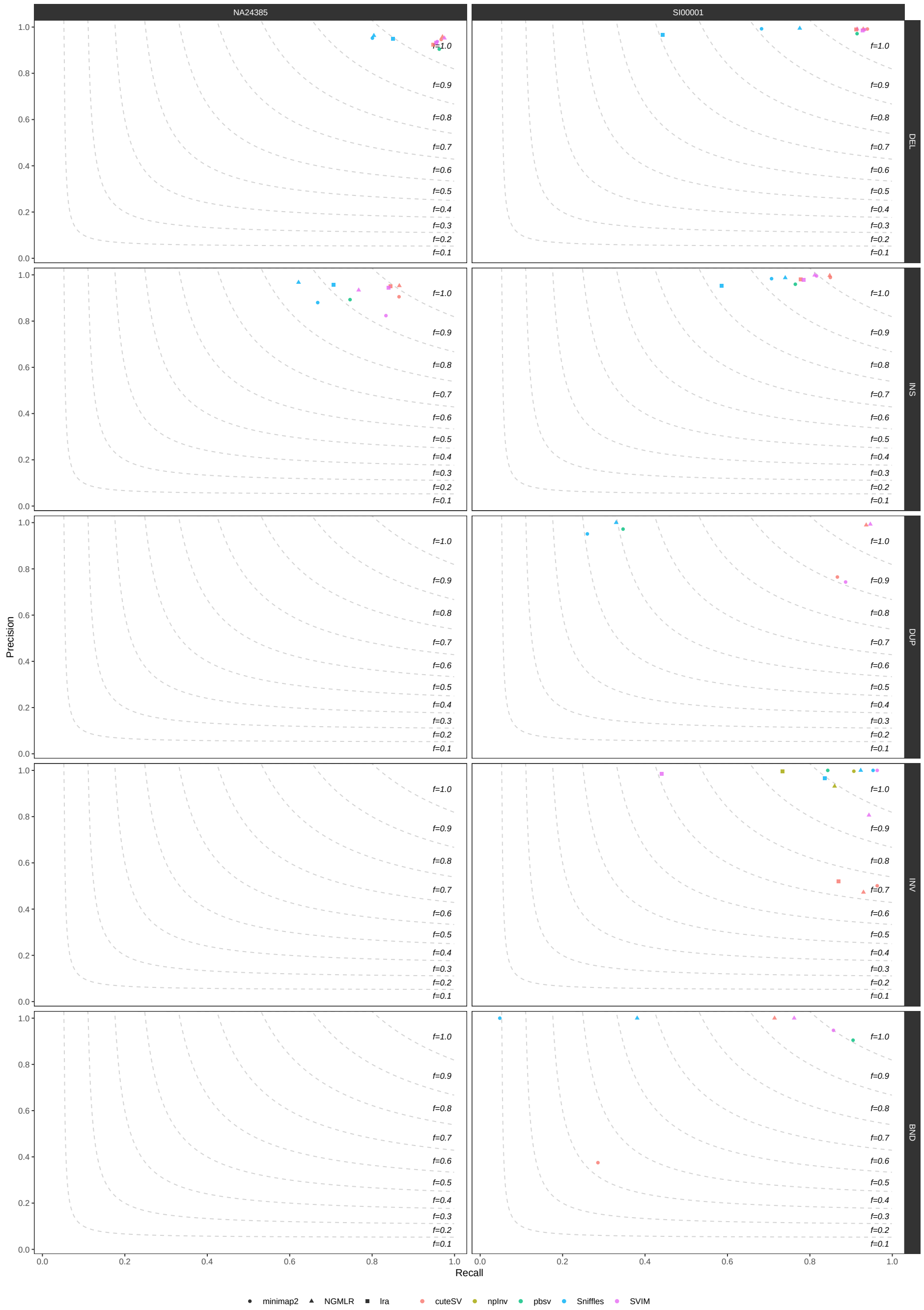

Figure S7: Precision (y axis), recall (x axis) and F-score (dashed lines) of the high-quality SV callsets from Sniffles, SVIM, cuteSV, npInv and pbsv (hue palette) after minimap2 (circle symbol), NGMLR (triangle symbol) and lra (square symbol) alignments, when resolved by SV type (deletions, insertions, duplications, inversions and translocations, top-to-bottom panels). Results are shown for the NA24385 dataset (left panels) and for the SI00001 dataset (right panels).

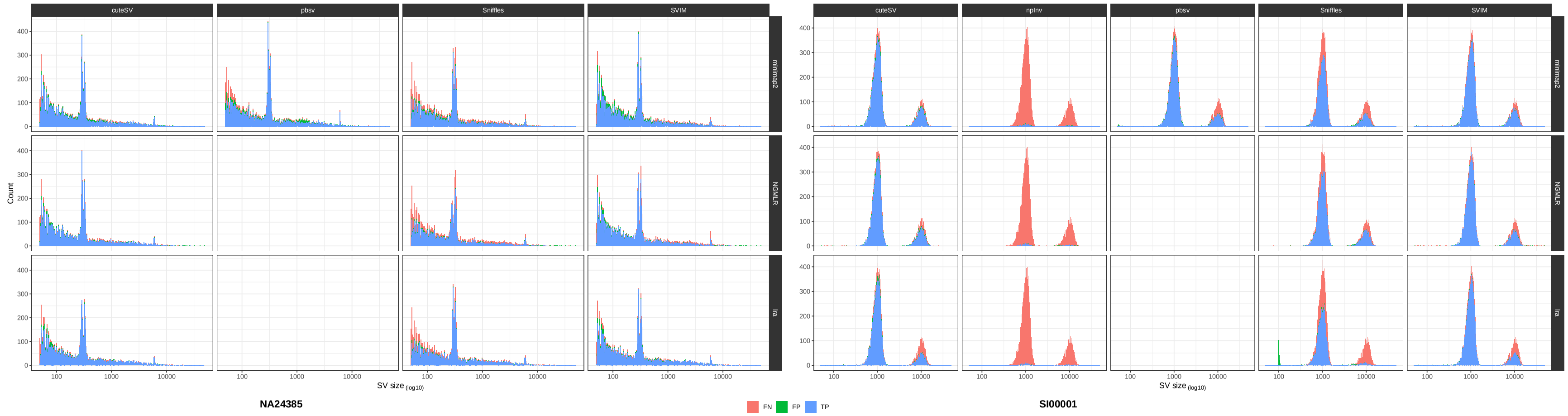

Figure S8: Size distribution of the true positive (TP), false positive (FP) and false negative (FN) high-quality SV calls (hue palette) from cuteSV, npInv, pbsv, Sniffles and SVIM (left-to-right sub-panels) after minimap2, NGMLR and lra alignments (top-to-bottom sub-panels). Results are shown for the NA24385 dataset (left panel) and for the SI00001 dataset (right panel).

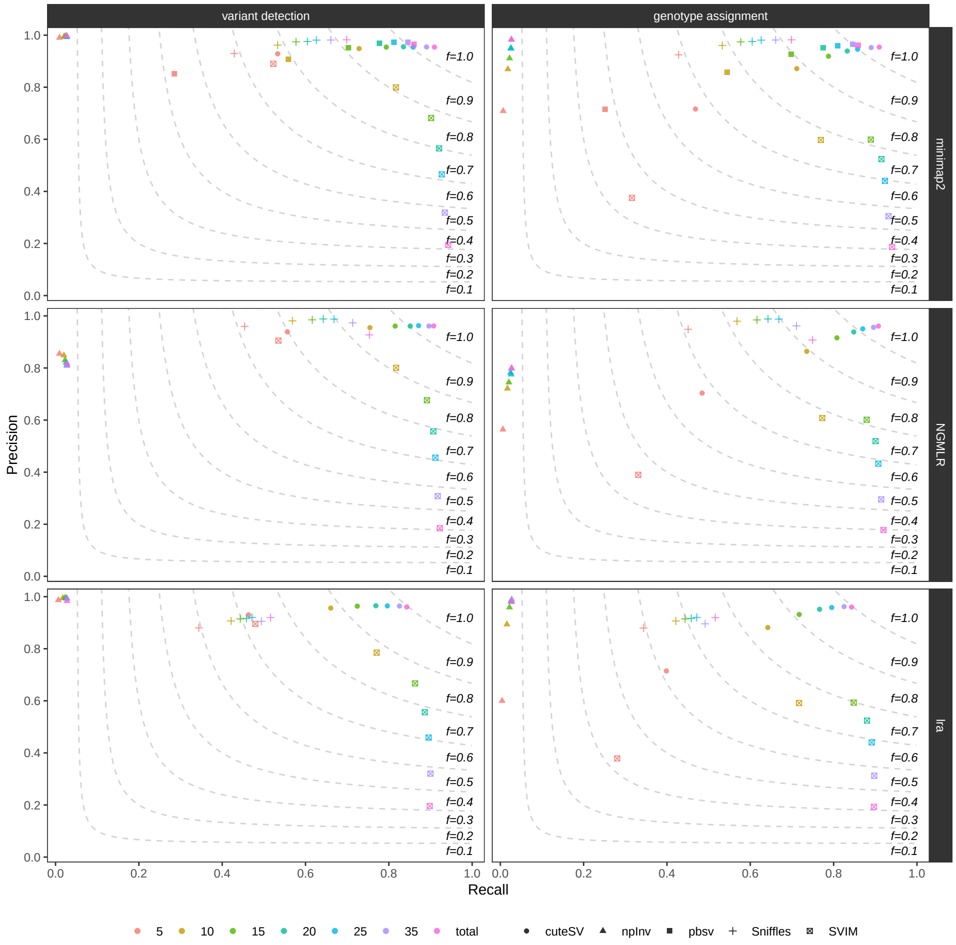

Figure S9: Precision (y axis), recall (x axis) and F-score (dashed lines) of the SV callers Sniffles (cross symbol), SVIM (square with cross symbol), cuteSV (circle symbol), pbsv (square symbol) and npInv (triangle symbol) after minimap2 (top panels), NGMLR mid panels) and lra (bottom panels) alignments. Results for both SV calling (left panels) and genotyping (right panels) are reported. The plot shows the influence of average genome coverage after down-sampling SI00001 alignments to different fractions (5X, 10X, 15X, 20X, 25X, 35X -hue palette) of the original coverage (total) on SV callers' performances.

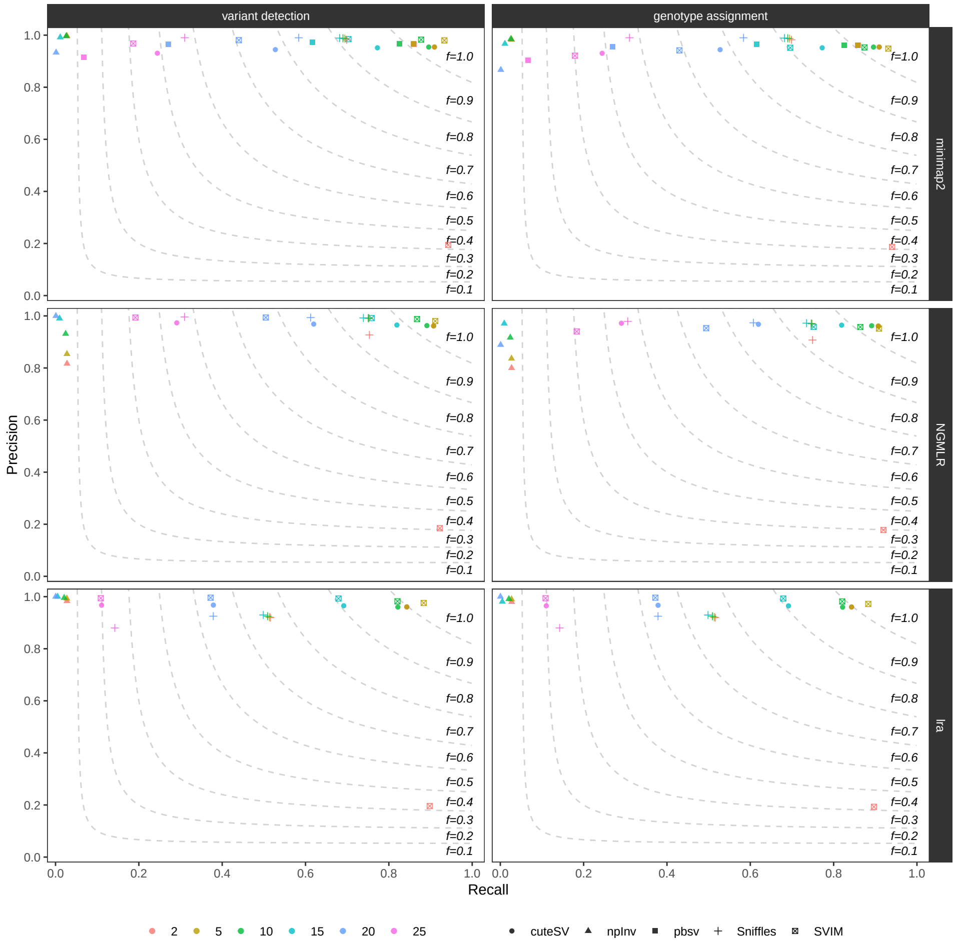

Figure S10: Precision (y axis), recall (x axis) and F-score (dashed lines) of the SV callers Sniffles (cross symbol), SVIM (square with cross symbol), cuteSV (circle symbol), pbsv (square symbol) and npInv (triangle symbol) after minimap2 (top panels), NGMLR (mid panels) and lra (bottom panels) alignments. Results for both SV calling (left panels) and genotyping (right panels) are reported. The plot shows the influence of the number of reads minimally supporting a SV (2,5,10,15,20,25 - hue palette) on SV callers' performances for the SI00001 dataset.

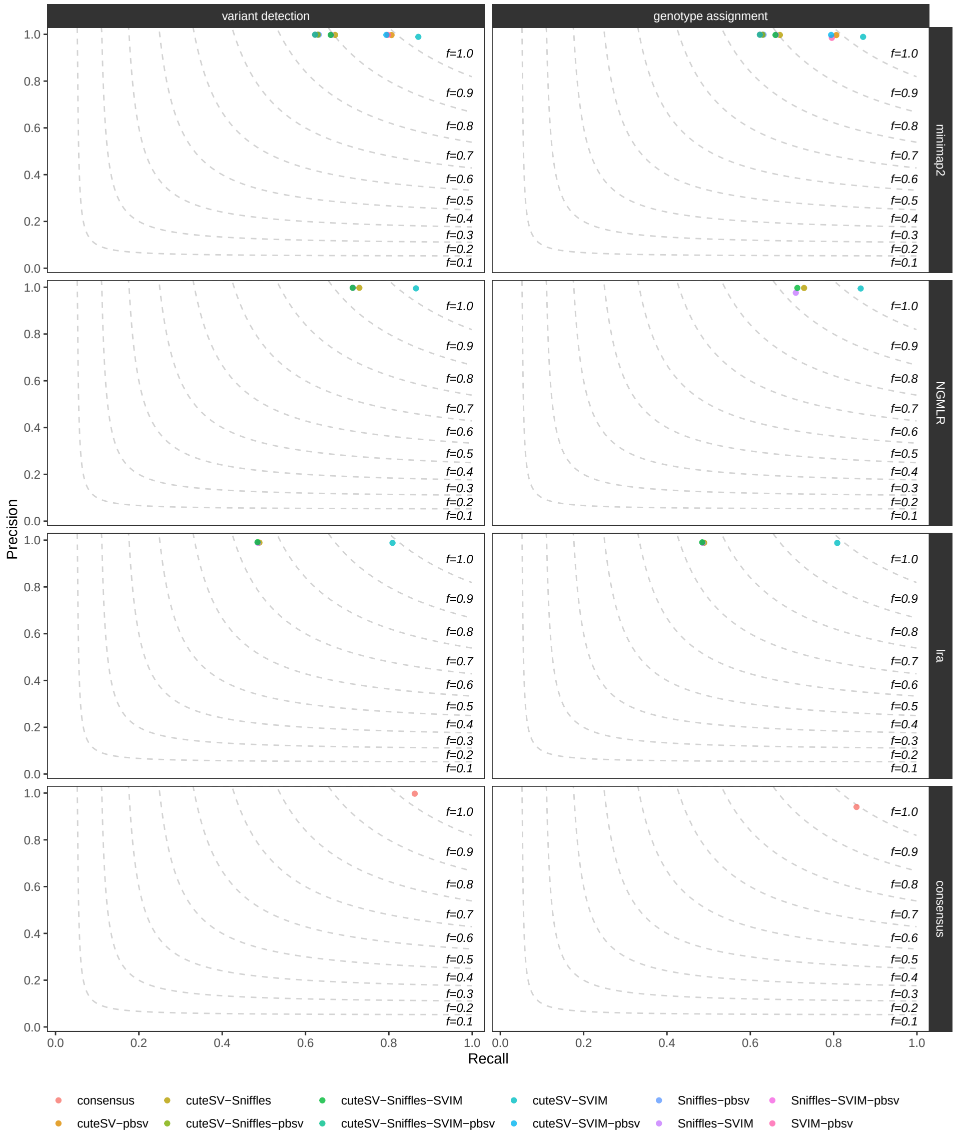

Figure S11: Precision (y axis), recall (x axis) and F-score (dashed lines) of the combination of the SV callers Sniffles, SVIM, cuteSV and pbsv (hue palette) after minimap2, NGMLR and lra alignments as well as after consensus generation (top-to-bottom panels). Results for both SV calling (left panels) and genotyping (right panels) are reported. The plot shows the influence of the integration of multiple high-quality callsets on reducing false positive calls in the SI00001 dataset.

Supplementary Tables

CALLSET	SIZE	DEL	INS	DUP	INV	TRA
	0-50	0	0	0	0	0
	50-100	2135	2155	0	0	0
	100-200	931	1357	0	0	0
	200-300	380	629	0	0	0
	300-400	1035	1287	0	0	0
NA24385	400-600	166	422	0	0	0
	600-800	109	313	0	0	0
	800-1k	90	178	0	0	0
	1k-2.5k	300	561	0	0	0
	2.5k-5k	162	226	0	0	0
	>=5k	156	153	0	0	0
	0-50	0	0	0	0	22
	50-100	0	0	0	0	0
	100-200	1	0	0	0	0
	200-300	0	0	0	0	0
	300-400	7	1	0	0	0
SI00001	400-600	86	100	2	0	0
	600-800	547	564	22	38	0
	800-1k	1335	1365	70	74	0
	1k-2.5k	2028	1974	116	98	0
	2.5k-5k	5	5	0	1	0
	>=5k	1018	1018	90	89	0

Table S 1: Size distribution of SVs in the NA24385 and SI00001 truth callsets, as reported by truvari. For translocations, all the translocated segments are ∼10000 bp-long in the SI00001 callset but, as truvari only considers a single breakpoint (1bp) when calculating statistics, they are all reported in the 0-50 bp size range.

TOOL	TYPE	VERSION	REPOSITORY	SETTING
minimap2	aligner	2.17	https://github.com/lh3/minimap2	minimap2 -ax map-ont
NGMLR	aligner	0.2.7	https://github.com/philres/ngmlr	ngmlr -x ont
lra	aligner	1.2.0	https://github.com/ChaissonLab/LRA	lra align -ONT
pbmm2	aligner	1.3.0	https://github.com/PacificBiosciences/pbmm2	pbmm2 align --preset subread[1]
Sniffles	SV caller	1.0.7	https://github.com/fritzsedlazeck/Sniffles	sniffles -s 2 -l 50
SVIM	SV caller	1.4.2	https://github.com/eldariont/svim	svim alignment --min_sv_size 50
cuteSV	SV caller	1.0.10	https://github.com/tjiangHIT/cuteSV	cuteSV -s 2 -l 50 --max_cluster_bias_DEL 100 --diff_ratio_merging_DEL 0.3
npInv	SV caller	1.24	https://github.com/haojingshao/npInv	npinv --min 50 --threshold 2
pbsv	SV caller	2.4.0	https://github.com/PacificBiosciences/pbsv	pbsv call -m 50

[1] As pbmm2 wraps minimap2, this is roughly equivalent to `minimap2 -k 19 -O 5,56 -E 4,1 -A 2 -B 5 -z 400,50 -r 2000 --lj-min-ratio 0.5`

Table S 2: List of the long-read aligners and SV callers tested in this paper. The table also includes the versions of the tools used, the corresponding repository and the minimal parameter settings used in our workflow.

CALLSET SV		ALIGNER & SV CALLER														
		minimap2					NGMLR					lra				
		Sniffles	SVIM	cuteSV	npInv	pbsv[1]	Sniffles	SVIM	cuteSV	npInv	pbsv[1]	Sniffles	SVIM	cuteSV	npInv	pbsv[1]
NA24385	DEL	25348(7033)	255393(9566)	38607(9450)	0	10941(9226)	25536(6545)	192531(9082)	27090(8755)	0	–	203745(7583)	2849325(9401)	221253(9456)	0	–
	INS	17698(8736)	198346(12818)	25437(11641)	0	13515(10218)	11840(6625)	133666(9619)	18588(10181)	0	–	24639(8544)	179680(10439)	33884(10948)	0	–
	DUP	1518(63)	7022(110)	8736(351)	0	2405(1941)	5996(778)	19125(1518)	12209(1735)	0	–	367(0)	2610(0)	606(0)	0	–
	INV	2307(69)	297(37)	629(121)	153(40)	106(83)	5077(75)	835(154)	1185(156)	407(44)	–	2368(2)	24471(10)	106(83)	128(0)	–
	BND[2]	5568(18)	41644(15)	8845(37)	0	3797(36)	12744(14)	55091(30)	6213(11)	0	–	426(0)	224(0)	589(0)	0	–
SI00001	DEL	20031(3458)	843513(4743)	21738(4763)	0	4922(4732)	23065(3920)	874939(4637)	23028(4713)	0	–	22226(2303)	839636(4738)	23205(4635)	0	–
	INS	15490(3614)	703739(4123)	16025(4320)	0	4267(4007)	15415(3769)	722068(4086)	16324(4277)	0	–	14741(3091)	657481(4032)	15352(3983)	0	–
	DUP	444(82)	583(358)	1084(340)	0	112(107)	1916(99)	1787(286)	834(284)	0	–	80(0)	167(0)	14(0)	0	–
	INV	328(286)	345(289)	617(577)	313(274)	253(253)	3364(277)	768(380)	1546(590)	612(278)	–	1235(260)	505(134)	868(501)	366(224)	–
	BND[2]	279(1)	264(34)	354(17)	0	52(39)	7246(12)	21840(32)	1111(24)	0	–	2949(0)	2253(0)	721(0)	0	–

[1] Pbsv is only tested in combination with the pbmm2 aligner
[2] Number of unique BNDs with breakpoints on different chromosomes

Table S 3: SVs identified by the different SV callers (Sniffles, SVIM, cuteSV, npInv and pbsv) after minimap2, NGMLR and lra alignments in the NA24385 and SI0001 dataset, further resolved by SV type. The table reports the number of SVs reported before and after filtering for high-quality SVs (*i.e.* SVs with the FILTER "PASS" that fall in assembled chromosomes only and are supported by ≥ 10 reads).

PRECISION	RECALL	F1	PRECISION_GT	RECALL_GT	F1_GT	TOOL	ALIGNER	CALLSET
0.9238	0.9099	0.9168	0.8877	0.9065	0.8970	cuteSV	minimap2	NA24385
0.9552	0.9116	0.9329	0.9183	0.9084	0.9133	cuteSV	NGMLR	NA24385
0.9379	0.8899	0.9133	0.8583	0.8810	0.8695	cuteSV	lra	NA24385
0.8734	0.8878	0.8805	0.7594	0.8731	0.8123	SVIM	minimap2	NA24385
0.9429	0.8580	0.8984	0.8416	0.8436	0.8426	SVIM	NGMLR	NA24385
0.9393	0.8902	0.9140	0.7234	0.8619	0.7866	SVIM	lra	NA24385
0.9132	0.7258	0.8088	0.4017	0.5379	0.4600	Sniffles	minimap2	NA24385
0.9653	0.7011	0.8122	0.4270	0.5092	0.4645	Sniffles	NGMLR	NA24385
0.9535	0.7691	0.8514	0.4506	0.6115	0.5188	Sniffles	lra	NA24385
0.8986	0.8407	0.8687	0.8272	0.8293	0.8282	pbsv	minimap2	NA24385
0.9543	0.8957	0.9241	0.9542	0.8957	0.9240	cuteSV	minimap2	SI00001
0.9627	0.8913	0.9256	0.9622	0.8913	0.9254	cuteSV	NGMLR	SI00001
0.9600	0.8217	0.8854	0.9596	0.8216	0.8853	cuteSV	lra	SI00001
0.9828	0.8775	0.9272	0.9529	0.8742	0.9118	SVIM	minimap2	SI00001
0.9877	0.8677	0.9239	0.9573	0.8641	0.9083	SVIM	NGMLR	SI00001
0.9820	0.8207	0.8942	0.9816	0.8207	0.8939	SVIM	lra	SI00001
0.9879	0.6899	0.8124	0.9879	0.6899	0.8124	Sniffles	minimap2	SI00001
0.9914	0.7504	0.8543	0.9705	0.7464	0.8438	Sniffles	NGMLR	SI00001
0.9248	0.5091	0.6567	0.9246	0.5091	0.6566	Sniffles	lra	SI00001
0.9963	0.0255	0.0498	0.9853	0.0253	0.0492	npInv	minimap2	SI00001
0.9314	0.0242	0.0472	0.9170	0.0238	0.0465	npInv	NGMLR	SI00001
0.9955	0.0206	0.0405	0.9910	0.0206	0.0403	npInv	lra	SI00001
0.9669	0.8258	0.8908	0.9615	0.8250	0.8880	pbsv	minimap2	SI00001

Table S 4: Precision, recall and F-score values (SV calling - column 1 to 3 - and genotyping - column 4 to 6 -) of the high-quality SV callsets (NA24385 and SI00001) from Sniffles, SVIM, cuteSV, npInv and pbsv after minimap2, NGMLR and lra alignments

PRECISION	RECALL	F1	PRECISION_GT	RECALL_GT	F1_GT	COVERAGE	TOOL	ALIGNER	CALLSET
0.8893	0.6972	0.7816	0.7393	0.6569	0.6956	5	cuteSV	minimap2	NA24385
0.8997	0.8321	0.8646	0.8121	0.8173	0.8147	10	cuteSV	minimap2	NA24385
0.9081	0.8697	0.8885	0.8437	0.8612	0.8524	15	cuteSV	minimap2	NA24385
0.9122	0.8890	0.9005	0.8602	0.8831	0.8715	20	cuteSV	minimap2	NA24385
0.9165	0.8987	0.9075	0.8708	0.8939	0.8822	25	cuteSV	minimap2	NA24385
0.9193	0.9098	0.9145	0.8803	0.9061	0.8930	35	cuteSV	minimap2	NA24385
0.9192	0.9143	0.9167	0.8814	0.9110	0.8960	total	cuteSV	minimap2	NA24385
0.9254	0.7075	0.8019	0.7790	0.6706	0.7208	5	cuteSV	NGMLR	NA24385
0.9282	0.8376	0.8805	0.8393	0.8234	0.8313	10	cuteSV	NGMLR	NA24385
0.9317	0.8790	0.9046	0.8670	0.8711	0.8690	15	cuteSV	NGMLR	NA24385
0.9369	0.9016	0.9189	0.8827	0.8961	0.8894	20	cuteSV	NGMLR	NA24385
0.9378	0.9139	0.9257	0.8903	0.9097	0.8999	25	cuteSV	NGMLR	NA24385
0.9381	0.9242	0.9311	0.8949	0.9208	0.9077	35	cuteSV	NGMLR	NA24385
0.9387	0.9271	0.9328	0.8959	0.9239	0.9097	total	cuteSV	NGMLR	NA24385
0.7680	0.6474	0.7026	0.5853	0.5833	0.5843	5	cuteSV	lra	NA24385
0.8851	0.7753	0.8266	0.7195	0.7372	0.7282	10	cuteSV	lra	NA24385
0.9211	0.8220	0.8687	0.7856	0.7975	0.7915	15	cuteSV	lra	NA24385
0.9289	0.8518	0.8886	0.8125	0.8341	0.8231	20	cuteSV	lra	NA24385
0.9316	0.8723	0.9010	0.8280	0.8586	0.8430	25	cuteSV	lra	NA24385
0.9343	0.8946	0.9140	0.8460	0.8849	0.8650	35	cuteSV	lra	NA24385
0.9344	0.9005	0.9171	0.8534	0.8921	0.8723	total	cuteSV	lra	NA24385
0.7916	0.7023	0.7443	0.4304	0.5620	0.4875	5	SVIM	minimap2	NA24385
0.6739	0.8810	0.7637	0.5252	0.8523	0.6499	10	SVIM	minimap2	NA24385
0.6092	0.9153	0.7315	0.5061	0.8997	0.6478	15	SVIM	minimap2	NA24385
0.5718	0.9154	0.7039	0.4834	0.9014	0.6293	20	SVIM	minimap2	NA24385
0.5425	0.9164	0.6816	0.4600	0.9029	0.6095	25	SVIM	minimap2	NA24385
0.4957	0.9165	0.6434	0.4219	0.9033	0.5752	35	SVIM	minimap2	NA24385
0.4577	0.9155	0.6102	0.3900	0.9022	0.5446	total	SVIM	minimap2	NA24385
0.8874	0.6762	0.7675	0.4955	0.5383	0.5160	5	SVIM	NGMLR	NA24385
0.7942	0.8544	0.8232	0.6261	0.8222	0.7109	10	SVIM	NGMLR	NA24385
0.7286	0.8923	0.8022	0.6153	0.8750	0.7225	15	SVIM	NGMLR	NA24385
0.6776	0.8974	0.7722	0.5874	0.8835	0.7056	20	SVIM	NGMLR	NA24385
0.6359	0.9005	0.7454	0.5546	0.8876	0.6827	25	SVIM	NGMLR	NA24385
0.5734	0.9006	0.7007	0.5040	0.8885	0.6431	35	SVIM	NGMLR	NA24385
0.5398	0.9021	0.6754	0.4730	0.8898	0.6176	total	SVIM	NGMLR	NA24385
0.5611	0.6967	0.6216	0.2660	0.5213	0.3522	5	SVIM	lra	NA24385
0.3150	0.8979	0.4663	0.2128	0.8560	0.3409	10	SVIM	lra	NA24385
0.1948	0.9340	0.3224	0.1437	0.9126	0.2484	15	SVIM	lra	NA24385
0.1317	0.9384	0.2309	0.0986	0.9194	0.1782	20	SVIM	lra	NA24385
0.0935	0.9374	0.1701	0.0704	0.9185	0.1308	25	SVIM	lra	NA24385
0.0557	0.9376	0.1052	0.0421	0.9190	0.0805	35	SVIM	lra	NA24385
0.0451	0.9365	0.0861	0.0341	0.9178	0.0658	total	SVIM	lra	NA24385
0.9195	0.5567	0.6935	0.3646	0.3324	0.3477	5	Sniffles	minimap2	NA24385
0.9074	0.6423	0.7521	0.3677	0.4211	0.3926	10	Sniffles	minimap2	NA24385
0.8803	0.6707	0.7613	0.3552	0.4511	0.3974	15	Sniffles	minimap2	NA24385
0.8319	0.6993	0.7599	0.3459	0.4916	0.4061	20	Sniffles	minimap2	NA24385
0.8167	0.7081	0.7586	0.3456	0.5066	0.4109	25	Sniffles	minimap2	NA24385
0.7784	0.7202	0.7481	0.3354	0.5258	0.4096	35	Sniffles	minimap2	NA24385
0.7289	0.7317	0.7303	0.3194	0.5444	0.4026	total	Sniffles	minimap2	NA24385
0.9505	0.5376	0.6868	0.4073	0.3325	0.3661	5	Sniffles	NGMLR	NA24385
0.9226	0.6208	0.7422	0.3854	0.4061	0.3955	10	Sniffles	NGMLR	NA24385
0.9116	0.6319	0.7464	0.3813	0.4179	0.3987	15	Sniffles	NGMLR	NA24385
0.8890	0.6537	0.7534	0.3755	0.4436	0.4067	20	Sniffles	NGMLR	NA24385
0.8590	0.6734	0.7549	0.3625	0.4653	0.4075	25	Sniffles	NGMLR	NA24385
0.7637	0.7001	0.7305	0.3287	0.5012	0.3970	35	Sniffles	NGMLR	NA24385
0.7319	0.7100	0.7208	0.3225	0.5189	0.3978	total	Sniffles	NGMLR	NA24385
0.7421	0.5134	0.6070	0.2844	0.2879	0.2862	5	Sniffles	lra	NA24385
0.9179	0.5731	0.7056	0.3590	0.3443	0.3515	10	Sniffles	lra	NA24385
0.6698	0.6308	0.6498	0.2602	0.3990	0.3150	15	Sniffles	lra	NA24385
0.4718	0.6884	0.5599	0.1944	0.4766	0.2762	20	Sniffles	lra	NA24385
0.5435	0.7187	0.6189	0.2332	0.5230	0.3225	25	Sniffles	lra	NA24385
0.4578	0.7654	0.5729	0.2074	0.5964	0.3078	35	Sniffles	lra	NA24385
0.3805	0.7734	0.5101	0.1794	0.6167	0.2780	total	Sniffles	lra	NA24385
0.8890	0.5392	0.6712	0.7638	0.5013	0.6053	5	pbsv	minimap2	NA24385
0.8968	0.7562	0.8206	0.8050	0.7358	0.7689	10	pbsv	minimap2	NA24385
0.9081	0.8214	0.8626	0.8250	0.8069	0.8158	15	pbsv	minimap2	NA24385
0.9091	0.8410	0.8737	0.8317	0.8287	0.8302	20	pbsv	minimap2	NA24385
0.9092	0.8478	0.8775	0.8331	0.8362	0.8347	25	pbsv	minimap2	NA24385
0.9010	0.8484	0.8739	0.8296	0.8374	0.8335	35	pbsv	minimap2	NA24385
0.8907	0.8484	0.8690	0.8190	0.8372	0.8280	total	pbsv	minimap2	NA24385

Continue

PRECISION	RECALL	F1	PRECISION_GT	RECALL_GT	F1_GT	COVERAGE	TOOL	ALIGNER	CALLSET
0.9284	0.5332	0.6774	0.7163	0.4685	0.5665	5	cuteSV	minimap2	SI00001
0.9484	0.7286	0.8241	0.8715	0.7116	0.7835	10	cuteSV	minimap2	SI00001
0.9540	0.7938	0.8665	0.9191	0.7876	0.8483	15	cuteSV	minimap2	SI00001
0.9552	0.8351	0.8911	0.9390	0.8327	0.8827	20	cuteSV	minimap2	SI00001
0.9538	0.8586	0.9037	0.9458	0.8575	0.8995	25	cuteSV	minimap2	SI00001
0.9547	0.8905	0.9215	0.9525	0.8902	0.9203	35	cuteSV	minimap2	SI00001
0.9542	0.9095	0.9313	0.9541	0.9095	0.9313	total	cuteSV	minimap2	SI00001
0.9390	0.5563	0.6987	0.7034	0.4843	0.5737	5	cuteSV	NGMLR	SI00001
0.9548	0.7546	0.8429	0.8638	0.7355	0.7945	10	cuteSV	NGMLR	SI00001
0.9608	0.8152	0.8820	0.9157	0.8078	0.8584	15	cuteSV	NGMLR	SI00001
0.9610	0.8514	0.9029	0.9382	0.8484	0.8910	20	cuteSV	NGMLR	SI00001
0.9627	0.8713	0.9147	0.9502	0.8699	0.9083	25	cuteSV	NGMLR	SI00001
0.9611	0.8963	0.9275	0.9561	0.8958	0.9250	35	cuteSV	NGMLR	SI00001
0.9613	0.9076	0.9337	0.9608	0.9076	0.9335	total	cuteSV	NGMLR	SI00001
0.9301	0.4632	0.6184	0.7147	0.3987	0.5118	5	cuteSV	lra	SI00001
0.9561	0.6605	0.7813	0.8814	0.6420	0.7429	10	cuteSV	lra	SI00001
0.9635	0.7242	0.8269	0.9314	0.7174	0.8105	15	cuteSV	lra	SI00001
0.9650	0.7688	0.8558	0.9515	0.7663	0.8489	20	cuteSV	lra	SI00001
0.9645	0.7964	0.8724	0.9583	0.7954	0.8693	25	cuteSV	lra	SI00001
0.9638	0.8254	0.8893	0.9617	0.8251	0.8882	35	cuteSV	lra	SI00001
0.9604	0.8431	0.8979	0.9601	0.8430	0.8978	total	cuteSV	lra	SI00001
0.8899	0.5228	0.6587	0.3750	0.3158	0.3429	5	SVIM	minimap2	SI00001
0.7994	0.8170	0.8081	0.5975	0.7694	0.6727	10	SVIM	minimap2	SI00001
0.6819	0.9014	0.7764	0.5990	0.8893	0.7158	15	SVIM	minimap2	SI00001
0.5652	0.9206	0.7004	0.5240	0.9149	0.6663	20	SVIM	minimap2	SI00001
0.4655	0.9271	0.6198	0.4404	0.9232	0.5964	25	SVIM	minimap2	SI00001
0.3184	0.9344	0.4750	0.3048	0.9317	0.4593	35	SVIM	minimap2	SI00001
0.1944	0.9426	0.3224	0.1867	0.9403	0.3116	total	SVIM	minimap2	SI00001
0.9053	0.5349	0.6725	0.3897	0.3311	0.3580	5	SVIM	NGMLR	SI00001
0.8007	0.8174	0.8090	0.6079	0.7727	0.6805	10	SVIM	NGMLR	SI00001
0.6761	0.8913	0.7689	0.6012	0.8794	0.7142	15	SVIM	NGMLR	SI00001
0.5567	0.9068	0.6899	0.5190	0.9007	0.6586	20	SVIM	NGMLR	SI00001
0.4556	0.9117	0.6075	0.4325	0.9074	0.5858	25	SVIM	NGMLR	SI00001
0.3080	0.9174	0.4611	0.2955	0.9142	0.4467	35	SVIM	NGMLR	SI00001
0.1847	0.9224	0.3078	0.1779	0.9196	0.2982	total	SVIM	NGMLR	SI00001
0.8955	0.4796	0.6247	0.3789	0.2806	0.3224	5	SVIM	lra	SI00001
0.7849	0.7707	0.7777	0.5915	0.7170	0.6482	10	SVIM	lra	SI00001
0.6670	0.8629	0.7524	0.5931	0.8484	0.6981	15	SVIM	lra	SI00001
0.5563	0.8863	0.6835	0.5240	0.8802	0.6569	20	SVIM	lra	SI00001
0.4589	0.8954	0.6068	0.4401	0.8915	0.5893	25	SVIM	lra	SI00001
0.3204	0.8998	0.4725	0.3123	0.8975	0.4634	35	SVIM	lra	SI00001
0.1960	0.8982	0.3217	0.1930	0.8968	0.3177	total	SVIM	lra	SI00001
0.9293	0.4291	0.5871	0.9246	0.4279	0.5850	5	Sniffles	minimap2	SI00001
0.9617	0.5330	0.6859	0.9605	0.5327	0.6854	10	Sniffles	minimap2	SI00001
0.9745	0.5772	0.7249	0.9742	0.5771	0.7248	15	Sniffles	minimap2	SI00001
0.9756	0.6046	0.7465	0.9756	0.6046	0.7465	20	Sniffles	minimap2	SI00001
0.9809	0.6261	0.7644	0.9809	0.6261	0.7644	25	Sniffles	minimap2	SI00001
0.9813	0.6609	0.7898	0.9813	0.6609	0.7898	35	Sniffles	minimap2	SI00001
0.9822	0.6988	0.8166	0.9822	0.6988	0.8166	total	Sniffles	minimap2	SI00001
0.9597	0.4537	0.6161	0.9486	0.4508	0.6112	5	Sniffles	NGMLR	SI00001
0.9811	0.5686	0.7199	0.9796	0.5682	0.7193	10	Sniffles	NGMLR	SI00001
0.9850	0.6162	0.7581	0.9845	0.6161	0.7579	15	Sniffles	NGMLR	SI00001
0.9882	0.6425	0.7787	0.9882	0.6425	0.7787	20	Sniffles	NGMLR	SI00001
0.9875	0.6684	0.7972	0.9875	0.6684	0.7972	25	Sniffles	NGMLR	SI00001
0.9732	0.7133	0.8233	0.9617	0.7109	0.8175	35	Sniffles	NGMLR	SI00001
0.9271	0.7533	0.8312	0.9075	0.7493	0.8209	total	Sniffles	NGMLR	SI00001
0.8799	0.3439	0.4945	0.8794	0.3438	0.4943	5	Sniffles	lra	SI00001
0.9067	0.4213	0.5753	0.9067	0.4213	0.5753	10	Sniffles	lra	SI00001
0.9145	0.4435	0.5973	0.9145	0.4435	0.5973	15	Sniffles	lra	SI00001
0.9166	0.4582	0.6110	0.9166	0.4582	0.6110	20	Sniffles	lra	SI00001
0.9203	0.4715	0.6235	0.9203	0.4715	0.6235	25	Sniffles	lra	SI00001
0.9053	0.4942	0.6393	0.8958	0.4916	0.6348	35	Sniffles	lra	SI00001
0.9195	0.5160	0.6610	0.9194	0.5159	0.6609	total	Sniffles	lra	SI00001
0.9903	0.0096	0.0190	0.7087	0.0069	0.0136	5	npInv	minimap2	SI00001
0.9955	0.0207	0.0406	0.8694	0.0182	0.0356	10	npInv	minimap2	SI00001
0.9961	0.0242	0.0473	0.9112	0.0222	0.0433	15	npInv	minimap2	SI00001
0.9929	0.0263	0.0512	0.9504	0.0252	0.0491	20	npInv	minimap2	SI00001
0.9931	0.0271	0.0528	0.9485	0.0259	0.0505	25	npInv	minimap2	SI00001
0.9965	0.0267	0.0519	0.9825	0.0263	0.0512	35	npInv	minimap2	SI00001
0.9966	0.0273	0.0532	0.9829	0.0269	0.0525	total	npInv	minimap2	SI00001
0.8547	0.0094	0.0186	0.5641	0.0062	0.0123	5	npInv	NGMLR	SI00001
0.8486	0.0200	0.0391	0.7211	0.0170	0.0333	10	npInv	NGMLR	SI00001
0.8299	0.0229	0.0446	0.7449	0.0206	0.0401	15	npInv	NGMLR	SI00001
0.8209	0.0258	0.0501	0.7821	0.0246	0.0477	20	npInv	NGMLR	SI00001
0.8118	0.0271	0.0525	0.7753	0.0259	0.0502	25	npInv	NGMLR	SI00001
0.8083	0.0273	0.0528	0.7972	0.0269	0.0521	35	npInv	NGMLR	SI00001
0.8167	0.0276	0.0534	0.8000	0.0270	0.0523	total	npInv	NGMLR	SI00001
0.9867	0.0069	0.0138	0.6000	0.0042	0.0084	5	npInv	lra	SI00001
0.9947	0.0176	0.0345	0.8936	0.0158	0.0310	10	npInv	lra	SI00001
0.9959	0.0228	0.0446	0.9590	0.0220	0.0430	15	npInv	lra	SI00001
0.9928	0.0258	0.0503	0.9819	0.0255	0.0498	20	npInv	lra	SI00001
0.9931	0.0269	0.0525	0.9827	0.0267	0.0519	25	npInv	lra	SI00001
0.9932	0.0275	0.0535	0.9898	0.0274	0.0533	35	npInv	lra	SI00001
0.9832	0.0274	0.0533	0.9798	0.0273	0.0532	total	npInv	lra	SI00001
0.8525	0.2853	0.4276	0.7156	0.2510	0.3717	5	pbsv	minimap2	SI00001
0.9066	0.5586	0.6913	0.8574	0.5448	0.6662	10	pbsv	minimap2	SI00001
0.9514	0.7033	0.8087	0.9264	0.6977	0.7959	15	pbsv	minimap2	SI00001
0.9688	0.7780	0.8630	0.9514	0.7749	0.8541	20	pbsv	minimap2	SI00001
0.9727	0.8117	0.8849	0.9598	0.8097	0.8784	25	pbsv	minimap2	SI00001
0.9731	0.8464	0.9054	0.9647	0.8453	0.9011	35	pbsv	minimap2	SI00001
0.9660	0.8601	0.9100	0.9607	0.8595	0.9073	total	pbsv	minimap2	SI00001

Table S 5: Precision, recall and F-score values (SV calling - column 1 to 3 - and genotyping - column 4 to 6 -) of the SV callsets (NA24385 and SI00001) from Sniffles, SVIM, cuteSV, npInv and pbsv after minimap2, NGMLR and lra alignments. Precision, recall and F1-score values are calculated for different coverage levels (5X, 10X, 15X, 20X, 25X, 35X and total coverage)

PRECISION	RECALL	F1	PRECISION_GT	RECALL_GT	F1_GT	SUPPORT	TOOL	ALIGNER	CALLSET
0.9192	0.9143	0.9167	0.8814	0.9110	0.8960	2	cuteSV	minimap2	NA24385
0.9207	0.9137	0.9172	0.8830	0.9104	0.8965	5	cuteSV	minimap2	NA24385
0.9238	0.9099	0.9168	0.8877	0.9065	0.8970	10	cuteSV	minimap2	NA24385
0.9339	0.8684	0.8999	0.8997	0.8641	0.8815	15	cuteSV	minimap2	NA24385
0.9402	0.7285	0.8209	0.9078	0.7215	0.8040	20	cuteSV	minimap2	NA24385
0.9417	0.5348	0.6822	0.9112	0.5266	0.6675	25	cuteSV	minimap2	NA24385
0.9407	0.4047	0.5660	0.9173	0.3987	0.5558	30	cuteSV	minimap2	NA24385
0.9415	0.3324	0.4914	0.9292	0.3295	0.4865	35	cuteSV	minimap2	NA24385
0.9483	0.2625	0.4112	0.9416	0.2611	0.4089	40	cuteSV	minimap2	NA24385
0.9496	0.1699	0.2882	0.9467	0.1695	0.2875	45	cuteSV	minimap2	NA24385
0.9468	0.0812	0.1496	0.9456	0.0811	0.1494	50	cuteSV	minimap2	NA24385
0.9387	0.9271	0.9328	0.8959	0.9239	0.9097	2	cuteSV	NGMLR	NA24385
0.9474	0.9231	0.9351	0.9071	0.9200	0.9135	5	cuteSV	NGMLR	NA24385
0.9552	0.9116	0.9329	0.9183	0.9084	0.9133	10	cuteSV	NGMLR	NA24385
0.9630	0.8493	0.9026	0.9287	0.8446	0.8847	15	cuteSV	NGMLR	NA24385
0.9731	0.6901	0.8075	0.9400	0.6826	0.7909	20	cuteSV	NGMLR	NA24385
0.9811	0.4907	0.6542	0.9504	0.4828	0.6403	25	cuteSV	NGMLR	NA24385
0.9846	0.3641	0.5316	0.9619	0.3587	0.5225	30	cuteSV	NGMLR	NA24385
0.9892	0.2951	0.4546	0.9750	0.2921	0.4495	35	cuteSV	NGMLR	NA24385
0.9881	0.2148	0.3529	0.9776	0.2130	0.3498	40	cuteSV	NGMLR	NA24385
0.9881	0.1288	0.2279	0.9801	0.1279	0.2263	45	cuteSV	NGMLR	NA24385
0.9928	0.0573	0.1083	0.9856	0.0569	0.1075	50	cuteSV	NGMLR	NA24385
0.9344	0.9005	0.9171	0.8534	0.8921	0.8723	2	cuteSV	lra	NA24385
0.9351	0.9000	0.9172	0.8542	0.8916	0.8725	5	cuteSV	lra	NA24385
0.9379	0.8899	0.9133	0.8583	0.8810	0.8695	10	cuteSV	lra	NA24385
0.9537	0.7728	0.8538	0.8710	0.7565	0.8097	15	cuteSV	lra	NA24385
0.9648	0.5597	0.7084	0.8736	0.5351	0.6637	20	cuteSV	lra	NA24385
0.9753	0.4010	0.5683	0.8925	0.3799	0.5330	25	cuteSV	lra	NA24385
0.9803	0.2991	0.4584	0.9283	0.2878	0.4394	30	cuteSV	lra	NA24385
0.9827	0.1949	0.3253	0.9592	0.1911	0.3187	35	cuteSV	lra	NA24385
0.9867	0.1004	0.1823	0.9817	0.0999	0.1814	40	cuteSV	lra	NA24385
0.9891	0.0378	0.0727	0.9864	0.0377	0.0725	45	cuteSV	lra	NA24385
1.0000	0.0090	0.0179	0.9885	0.0089	0.0177	50	cuteSV	lra	NA24385
0.4577	0.9155	0.6102	0.3900	0.9022	0.5446	2	SVIM	minimap2	NA24385
0.8450	0.9015	0.8723	0.7322	0.8880	0.8026	5	SVIM	minimap2	NA24385
0.8734	0.8878	0.8805	0.7594	0.8731	0.8123	10	SVIM	minimap2	NA24385
0.9332	0.8182	0.8719	0.8165	0.7975	0.8069	15	SVIM	minimap2	NA24385
0.9607	0.6652	0.7861	0.8390	0.6343	0.7224	20	SVIM	minimap2	NA24385
0.9739	0.4762	0.6396	0.8420	0.4401	0.5780	25	SVIM	minimap2	NA24385
0.9818	0.3471	0.5128	0.8606	0.3178	0.4642	30	SVIM	minimap2	NA24385
0.9850	0.2727	0.4271	0.9060	0.2564	0.3997	35	SVIM	minimap2	NA24385
0.9880	0.2053	0.3399	0.9481	0.1986	0.3284	40	SVIM	minimap2	NA24385
0.9912	0.1284	0.2274	0.9728	0.1263	0.2236	45	SVIM	minimap2	NA24385
0.9882	0.0607	0.1143	0.9797	0.0602	0.1134	50	SVIM	minimap2	NA24385
0.5398	0.9021	0.6754	0.4730	0.8898	0.6176	2	SVIM	NGMLR	NA24385
0.9304	0.8868	0.9081	0.8297	0.8748	0.8517	5	SVIM	NGMLR	NA24385
0.9429	0.8580	0.8984	0.8416	0.8436	0.8426	10	SVIM	NGMLR	NA24385
0.9651	0.7493	0.8436	0.8635	0.7278	0.7899	15	SVIM	NGMLR	NA24385
0.9772	0.5879	0.7341	0.8716	0.5599	0.6818	20	SVIM	NGMLR	NA24385
0.9856	0.4130	0.5821	0.8782	0.3854	0.5357	25	SVIM	NGMLR	NA24385
0.9912	0.3047	0.4662	0.9072	0.2863	0.4353	30	SVIM	NGMLR	NA24385
0.9936	0.2418	0.3889	0.9386	0.2315	0.3714	35	SVIM	NGMLR	NA24385
0.9934	0.1706	0.2912	0.9686	0.1671	0.2850	40	SVIM	NGMLR	NA24385
0.9918	0.1003	0.1822	0.9785	0.0991	0.1799	45	SVIM	NGMLR	NA24385
0.9953	0.0441	0.0844	0.9930	0.0440	0.0842	50	SVIM	NGMLR	NA24385
0.0451	0.9365	0.0861	0.0341	0.9178	0.0658	2	SVIM	lra	NA24385
0.9197	0.9224	0.9211	0.7096	0.9017	0.7942	5	SVIM	lra	NA24385
0.9393	0.8902	0.9140	0.7234	0.8619	0.7866	10	SVIM	lra	NA24385
0.9685	0.7345	0.8354	0.7247	0.6742	0.6985	15	SVIM	lra	NA24385
0.9800	0.5136	0.6740	0.6838	0.4242	0.5236	20	SVIM	lra	NA24385
0.9888	0.3572	0.5248	0.6629	0.2715	0.3852	25	SVIM	lra	NA24385
0.9911	0.2543	0.4048	0.7243	0.1995	0.3129	30	SVIM	lra	NA24385
0.9920	0.1542	0.2670	0.8212	0.1312	0.2262	35	SVIM	lra	NA24385
0.9946	0.0765	0.1422	0.9178	0.0711	0.1319	40	SVIM	lra	NA24385
0.9925	0.0275	0.0535	0.9700	0.0269	0.0523	45	SVIM	lra	NA24385
1.0000	0.0065	0.0130	0.9841	0.0064	0.0128	50	SVIM	lra	NA24385
0.7289	0.7317	0.7303	0.3194	0.5444	0.4026	2	Sniffles	minimap2	NA24385
0.8889	0.7297	0.8015	0.3898	0.5421	0.4535	5	Sniffles	minimap2	NA24385
0.9132	0.7258	0.8088	0.4017	0.5379	0.4600	10	Sniffles	minimap2	NA24385
0.9187	0.7123	0.8024	0.4079	0.5236	0.4586	15	Sniffles	minimap2	NA24385
0.9212	0.6474	0.7604	0.4008	0.4442	0.4214	20	Sniffles	minimap2	NA24385
0.9189	0.4934	0.6421	0.3284	0.2582	0.2891	25	Sniffles	minimap2	NA24385
0.9166	0.3695	0.5267	0.2385	0.1323	0.1702	30	Sniffles	minimap2	NA24385
0.9196	0.3014	0.4540	0.1968	0.0845	0.1183	35	Sniffles	minimap2	NA24385
0.9321	0.2350	0.3754	0.1954	0.0605	0.0924	40	Sniffles	minimap2	NA24385
0.9379	0.1504	0.2592	0.1947	0.0354	0.0600	45	Sniffles	minimap2	NA24385
0.9432	0.0723	0.1343	0.2016	0.0164	0.0303	50	Sniffles	minimap2	NA24385
0.7319	0.7100	0.7208	0.3225	0.5189	0.3978	2	Sniffles	NGMLR	NA24385
0.9315	0.7069	0.8038	0.4105	0.5152	0.4569	5	Sniffles	NGMLR	NA24385
0.9653	0.7011	0.8122	0.4270	0.5092	0.4645	10	Sniffles	NGMLR	NA24385
0.9699	0.6793	0.7990	0.4365	0.4880	0.4608	15	Sniffles	NGMLR	NA24385
0.9724	0.5928	0.7366	0.4284	0.3908	0.4087	20	Sniffles	NGMLR	NA24385
0.9754	0.4324	0.5992	0.3617	0.2203	0.2738	25	Sniffles	NGMLR	NA24385
0.9753	0.3241	0.4866	0.2856	0.1231	0.1721	30	Sniffles	NGMLR	NA24385
0.9790	0.2560	0.4059	0.2622	0.0844	0.1277	35	Sniffles	NGMLR	NA24385
0.9811	0.1830	0.3084	0.2597	0.0560	0.0921	40	Sniffles	NGMLR	NA24385
0.9793	0.1030	0.1864	0.2485	0.0283	0.0508	45	Sniffles	NGMLR	NA24385
0.9745	0.0396	0.0761	0.2730	0.0114	0.0219	50	Sniffles	NGMLR	NA24385

Continue

PRECISION	RECALL	F1	PRECISION_GT	RECALL_GT	F1_GT	SUPPORT	TOOL	ALIGNER	CALLSET
0.3805	0.7734	0.5101	0.1794	0.6167	0.2780	2	Sniffles	lra	NA24385
0.9408	0.7721	0.8482	0.4439	0.6152	0.5157	5	Sniffles	lra	NA24385
0.9535	0.7691	0.8514	0.4506	0.6115	0.5188	10	Sniffles	lra	NA24385
0.9574	0.7407	0.8352	0.4431	0.5693	0.4983	15	Sniffles	lra	NA24385
0.9589	0.5885	0.7294	0.3439	0.3391	0.3415	20	Sniffles	lra	NA24385
0.9635	0.4238	0.5887	0.1745	0.1176	0.1405	25	Sniffles	lra	NA24385
0.9678	0.3246	0.4861	0.0872	0.0415	0.0562	30	Sniffles	lra	NA24385
0.9699	0.2271	0.3680	0.0616	0.0183	0.0282	35	Sniffles	lra	NA24385
0.9710	0.1249	0.2213	0.0589	0.0086	0.0150	40	Sniffles	lra	NA24385
0.9756	0.0497	0.0946	0.0652	0.0035	0.0066	45	Sniffles	lra	NA24385
0.9779	0.0138	0.0272	0.0809	0.0012	0.0023	50	Sniffles	lra	NA24385
0.8907	0.8484	0.8690	0.8190	0.8372	0.8280	2	pbsv	minimap2	NA24385
0.8909	0.8481	0.8690	0.8194	0.8370	0.8281	5	pbsv	minimap2	NA24385
0.8986	0.8407	0.8687	0.8272	0.8293	0.8282	10	pbsv	minimap2	NA24385
0.9210	0.7877	0.8492	0.8480	0.7735	0.8091	15	pbsv	minimap2	NA24385
0.9388	0.6400	0.7611	0.8554	0.6183	0.7178	20	pbsv	minimap2	NA24385
0.9487	0.4584	0.6181	0.8491	0.4310	0.5717	25	pbsv	minimap2	NA24385
0.9581	0.3486	0.5112	0.8709	0.3273	0.4757	30	pbsv	minimap2	NA24385
0.9656	0.2763	0.4297	0.9047	0.2635	0.4081	35	pbsv	minimap2	NA24385
0.9711	0.1918	0.3203	0.9338	0.1858	0.3099	40	pbsv	minimap2	NA24385
0.9709	0.1075	0.1935	0.9550	0.1059	0.1906	45	pbsv	minimap2	NA24385
0.9601	0.0449	0.0858	0.9534	0.0446	0.0852	50	pbsv	minimap2	NA24385
0.9542	0.9095	0.9313	0.9541	0.9095	0.9313	2	cuteSV	minimap2	SI00001
0.9546	0.9094	0.9315	0.9545	0.9094	0.9314	5	cuteSV	minimap2	SI00001
0.9543	0.8957	0.9241	0.9542	0.8957	0.9240	10	cuteSV	minimap2	SI00001
0.9515	0.7726	0.8527	0.9513	0.7726	0.8527	15	cuteSV	minimap2	SI00001
0.9444	0.5276	0.6770	0.9442	0.5276	0.6769	20	cuteSV	minimap2	SI00001
0.9307	0.2444	0.3872	0.9303	0.2443	0.3870	25	cuteSV	minimap2	SI00001
0.9613	0.9076	0.9337	0.9608	0.9076	0.9335	2	cuteSV	NGMLR	SI00001
0.9617	0.9073	0.9337	0.9612	0.9072	0.9334	5	cuteSV	NGMLR	SI00001
0.9627	0.8913	0.9256	0.9622	0.8913	0.9254	10	cuteSV	NGMLR	SI00001
0.9650	0.8193	0.8862	0.9644	0.8192	0.8859	15	cuteSV	NGMLR	SI00001
0.9686	0.6197	0.7558	0.9679	0.6195	0.7555	20	cuteSV	NGMLR	SI00001
0.9733	0.2910	0.4480	0.9717	0.2906	0.4474	25	cuteSV	NGMLR	SI00001
0.9604	0.8431	0.8979	0.9601	0.8430	0.8978	2	cuteSV	lra	SI00001
0.9606	0.8430	0.8980	0.9603	0.8429	0.8978	5	cuteSV	lra	SI00001
0.9600	0.8217	0.8854	0.9596	0.8216	0.8853	10	cuteSV	lra	SI00001
0.9650	0.6918	0.8059	0.9646	0.6917	0.8057	15	cuteSV	lra	SI00001
0.9676	0.3788	0.5445	0.9669	0.3786	0.5442	20	cuteSV	lra	SI00001
0.9672	0.1106	0.1985	0.9647	0.1103	0.1980	25	cuteSV	lra	SI00001
0.1944	0.9426	0.3224	0.1867	0.9403	0.3116	2	SVIM	minimap2	SI00001
0.9799	0.9334	0.9561	0.9484	0.9313	0.9398	5	SVIM	minimap2	SI00001
0.9828	0.8775	0.9272	0.9529	0.8742	0.9118	10	SVIM	minimap2	SI00001
0.9847	0.7030	0.8204	0.9516	0.6958	0.8039	15	SVIM	minimap2	SI00001
0.9807	0.4399	0.6074	0.9416	0.4299	0.5903	20	SVIM	minimap2	SI00001
0.9679	0.1865	0.3127	0.9211	0.1791	0.2999	25	SVIM	minimap2	SI00001
0.1847	0.9224	0.3078	0.1779	0.9196	0.2982	2	SVIM	NGMLR	SI00001
0.9802	0.9114	0.9446	0.9513	0.9090	0.9297	5	SVIM	NGMLR	SI00001
0.9877	0.8677	0.9239	0.9573	0.8641	0.9083	10	SVIM	NGMLR	SI00001
0.9917	0.7586	0.8596	0.9577	0.7521	0.8426	15	SVIM	NGMLR	SI00001
0.9939	0.5047	0.6694	0.9529	0.4942	0.6508	20	SVIM	NGMLR	SI00001
0.9937	0.1918	0.3215	0.9402	0.1833	0.3068	25	SVIM	NGMLR	SI00001
0.1960	0.8982	0.3217	0.1930	0.8968	0.3177	2	SVIM	lra	SI00001
0.9756	0.8836	0.9274	0.9719	0.8832	0.9254	5	SVIM	lra	SI00001
0.9820	0.8207	0.8942	0.9816	0.8207	0.8939	10	SVIM	lra	SI00001
0.9929	0.6792	0.8066	0.9929	0.6792	0.8066	15	SVIM	lra	SI00001
0.9960	0.3722	0.5419	0.9960	0.3722	0.5419	20	SVIM	lra	SI00001
0.9940	0.1086	0.1958	0.9940	0.1086	0.1958	25	SVIM	lra	SI00001
0.9822	0.6988	0.8166	0.9822	0.6988	0.8166	2	Sniffles	minimap2	SI00001
0.9867	0.6943	0.8151	0.9867	0.6943	0.8151	5	Sniffles	minimap2	SI00001
0.9879	0.6899	0.8124	0.9879	0.6899	0.8124	10	Sniffles	minimap2	SI00001
0.9887	0.6820	0.8072	0.9887	0.6820	0.8072	15	Sniffles	minimap2	SI00001
0.9900	0.5837	0.7344	0.9900	0.5837	0.7344	20	Sniffles	minimap2	SI00001
0.9901	0.3100	0.4722	0.9901	0.3100	0.4722	25	Sniffles	minimap2	SI00001
0.9271	0.7533	0.8312	0.9075	0.7493	0.8209	2	Sniffles	NGMLR	SI00001
0.9909	0.7523	0.8553	0.9698	0.7483	0.8448	5	Sniffles	NGMLR	SI00001
0.9914	0.7504	0.8543	0.9705	0.7464	0.8438	10	Sniffles	NGMLR	SI00001
0.9923	0.7389	0.8470	0.9721	0.7349	0.8370	15	Sniffles	NGMLR	SI00001
0.9936	0.6123	0.7577	0.9735	0.6074	0.7481	20	Sniffles	NGMLR	SI00001
0.9961	0.3096	0.4724	0.9789	0.3059	0.4662	25	Sniffles	NGMLR	SI00001
0.9195	0.5160	0.6610	0.9194	0.5159	0.6609	2	Sniffles	lra	SI00001
0.9212	0.5134	0.6594	0.9210	0.5134	0.6593	5	Sniffles	lra	SI00001
0.9248	0.5091	0.6567	0.9246	0.5091	0.6566	10	Sniffles	lra	SI00001
0.9298	0.4986	0.6491	0.9296	0.4985	0.6490	15	Sniffles	lra	SI00001
0.9250	0.3785	0.5372	0.9248	0.3785	0.5371	20	Sniffles	lra	SI00001
0.8799	0.1423	0.2450	0.8799	0.1423	0.2450	25	Sniffles	lra	SI00001

Continue

PRECISION	RECALL	F1	PRECISION_GT	RECALL_GT	F1_GT	SUPPORT	TOOL	ALIGNER	CALLSET
0.9966	0.0273	0.0532	0.9829	0.0269	0.0525	2	npInv	minimap2	SI00001
0.9966	0.0272	0.0530	0.9828	0.0269	0.0523	5	npInv	minimap2	SI00001
0.9963	0.0255	0.0498	0.9853	0.0253	0.0492	10	npInv	minimap2	SI00001
0.9917	0.0113	0.0223	0.9669	0.0110	0.0217	15	npInv	minimap2	SI00001
0.9333	0.0013	0.0026	0.8667	0.0012	0.0024	20	npInv	minimap2	SI00001
0.0000	0.0000	NaN	0.0000	0.0000	NaN	25	npInv	minimap2	SI00001
0.8167	0.0276	0.0534	0.8000	0.0270	0.0523	2	npInv	NGMLR	SI00001
0.8538	0.0274	0.0531	0.8363	0.0269	0.0520	5	npInv	NGMLR	SI00001
0.9314	0.0242	0.0472	0.9170	0.0238	0.0465	10	npInv	NGMLR	SI00001
0.9903	0.0096	0.0190	0.9709	0.0094	0.0186	15	npInv	NGMLR	SI00001
1.0000	0.0008	0.0017	0.8889	0.0008	0.0015	20	npInv	NGMLR	SI00001
0.0000	0.0000	NaN	0.0000	0.0000	NaN	25	npInv	NGMLR	SI00001
0.9832	0.0274	0.0533	0.9798	0.0273	0.0532	2	npInv	lra	SI00001
0.9931	0.0271	0.0528	0.9897	0.0270	0.0526	5	npInv	lra	SI00001
0.9955	0.0206	0.0405	0.9910	0.0206	0.0403	10	npInv	lra	SI00001
1.0000	0.0049	0.0097	0.9808	0.0048	0.0095	15	npInv	lra	SI00001
1.0000	0.0003	0.0006	1.0000	0.0003	0.0006	20	npInv	lra	SI00001
0.0000	0.0000	NaN	0.0000	0.0000	NaN	25	npInv	lra	SI00001
0.9660	0.8601	0.9100	0.9607	0.8595	0.9073	2	pbsv	minimap2	SI00001
0.9663	0.8586	0.9092	0.9611	0.8579	0.9066	5	pbsv	minimap2	SI00001
0.9669	0.8258	0.8908	0.9615	0.8250	0.8880	10	pbsv	minimap2	SI00001
0.9719	0.6170	0.7548	0.9647	0.6152	0.7513	15	pbsv	minimap2	SI00001
0.9659	0.2711	0.4233	0.9552	0.2689	0.4196	20	pbsv	minimap2	SI00001
0.9149	0.0676	0.1259	0.9034	0.0668	0.1244	25	pbsv	minimap2	SI00001

Table S 6: Precision, recall and F-score values (SV calling - column 1 to 3 - and genotyping - column 4 to 6 -) of the SV callsets (NA24385 and SI00001) from Sniffles, SVIM, cuteSV, npInv and pbsv after minimap2, NGMLR and lra alignments. Precision, recall and F-score values are calculated for different minimum number of reads supporting a SV (2,5,10,15,20,25,30,35,40,45,50 - for the NA24385 dataset - and 5,10,15,20,25 - for the SI00001 dataset).

PRECISION	RECALL	F1	PRECISION_GT	RECALL_GT	F1_GT	COMBINATION	ALIGNER	CALLSET
0.9381	0.7298	0.8210	0.9112	0.7240	0.8069	cutesv-sniffles	minimap2	NA24385
0.9746	0.7010	0.8154	0.9456	0.6946	0.8009	cutesv-sniffles	NGMLR	NA24385
0.9642	0.7647	0.8529	0.8856	0.7490	0.8116	cutesv-sniffles	lra	NA24385
0.9456	0.8770	0.9100	0.9106	0.8729	0.8913	cutesv-svim	minimap2	NA24385
0.9659	0.8471	0.9026	0.9284	0.8419	0.8831	cutesv-svim	NGMLR	NA24385
0.9621	0.8718	0.9147	0.8822	0.8618	0.8719	cutesv-svim	lra	NA24385
0.9567	0.8211	0.8837	0.9362	0.8179	0.8730	cutesv-pbsv	minimap2	NA24385
0.9552	0.6849	0.7978	0.4199	0.4886	0.4517	sniffles-pbsv	minimap2	NA24385
0.9567	0.7991	0.8708	0.8491	0.7793	0.8127	svim-pbsv	minimap2	NA24385
0.9458	0.7210	0.8182	0.4138	0.5306	0.4650	sniffles-svim	minimap2	NA24385
0.9755	0.6856	0.8053	0.4289	0.4895	0.4572	sniffles-svim	NGMLR	NA24385
0.9693	0.7565	0.8498	0.4552	0.5933	0.5152	sniffles-svim	lra	NA24385
0.9528	0.7222	0.8216	0.9252	0.7163	0.8074	cutesv-sniffles-svim	minimap2	NA24385
0.9776	0.6845	0.8052	0.9483	0.6779	0.7906	cutesv-sniffles-svim	NGMLR	NA24385
0.9717	0.7542	0.8492	0.8944	0.7385	0.8090	cutesv-sniffles-svim	lra	NA24385
0.9606	0.6851	0.7998	0.9417	0.6808	0.7903	cutesv-sniffles-pbsv	minimap2	NA24385
0.9630	0.7961	0.8716	0.9422	0.7925	0.8609	cutesv-svim-pbsv	minimap2	NA24385
0.9636	0.6749	0.7938	0.4232	0.4770	0.4485	sniffles-svim-pbsv	minimap2	NA24385
0.9657	0.6740	0.7939	0.9465	0.6696	0.7843	cutesv-sniffles-svim-pbsv	minimap2	NA24385
0.9659	0.8930	0.9280	0.8613	0.8815	0.8713	consensus	consensus	NA24385
0.9973	0.6710	0.8023	0.9972	0.6710	0.8022	cutesv-sniffles	minimap2	SI00001
0.9974	0.7292	0.8425	0.9968	0.7291	0.8422	cutesv-sniffles	NGMLR	SI00001
0.9896	0.4894	0.6549	0.9890	0.4892	0.6546	cutesv-sniffles	lra	SI00001
0.9891	0.8708	0.9262	0.9890	0.8707	0.9261	cutesv-svim	minimap2	SI00001
0.9951	0.8651	0.9256	0.9946	0.8651	0.9253	cutesv-svim	NGMLR	SI00001
0.9883	0.8088	0.8896	0.9880	0.8088	0.8894	cutesv-svim	lra	SI00001
0.9968	0.8065	0.8916	0.9966	0.8064	0.8915	cutesv-pbsv	minimap2	SI00001
0.9984	0.6319	0.7739	0.9984	0.6319	0.7739	sniffles-pbsv	minimap2	SI00001
0.9974	0.7977	0.8865	0.9849	0.7957	0.8802	svim-pbsv	minimap2	SI00001
0.9973	0.6618	0.7956	0.9973	0.6618	0.7956	sniffles-svim	minimap2	SI00001
0.9975	0.7136	0.8320	0.9763	0.7092	0.8216	sniffles-svim	NGMLR	SI00001
0.9885	0.4858	0.6515	0.9883	0.4858	0.6514	sniffles-svim	lra	SI00001
0.9973	0.6605	0.7947	0.9972	0.6605	0.7946	cutesv-sniffles-svim	minimap2	SI00001
0.9975	0.7132	0.8317	0.9968	0.7130	0.8314	cutesv-sniffles-svim	NGMLR	SI00001
0.9912	0.4848	0.6511	0.9906	0.4846	0.6509	cutesv-sniffles-svim	lra	SI00001
0.9984	0.6293	0.7720	0.9982	0.6293	0.7720	cutesv-sniffles-pbsv	minimap2	SI00001
0.9975	0.7941	0.8842	0.9974	0.7940	0.8842	cutesv-svim-pbsv	minimap2	SI00001
0.9983	0.6231	0.7673	0.9983	0.6231	0.7673	sniffles-svim-pbsv	minimap2	SI00001
0.9983	0.6230	0.7672	0.9982	0.6229	0.7671	cutesv-sniffles-svim-pbsv	minimap2	SI00001
0.9973	0.8621	0.9248	0.9405	0.8550	0.8957	consensus	consensus	SI00001

Table S 7: Precision, recall and F-score values (SV calling - column 1 to 3 - and genotyping - column 4 to 6 -) of the combined callsets from Sniffles, SVIM, cuteSV and pbsv after minimap2, NGMLR and lra alignments.

References

[1] Mircea Cretu Stancu et al. "Mapping and phasing of structural variation in patient genomes using nanopore sequencing". In: *Nature Communications* 8.1 (Nov. 2017), p. 1326. ISSN: 2041-1723. DOI: 10.1038/s41467-017-01343-4. URL: https://doi.org/10.1038/s41467-017-01343-4.

[2] Liang Gong et al. "Picky comprehensively detects high-resolution structural variants in nanopore long reads". In: *Nature Methods* 15.6 (June 2018), pp. 455–460. ISSN: 1548-7105. DOI: 10.1038/s41592-018-0002-6. URL: https://doi.org/10.1038/s41592-018-0002-6.

[3] David Heller and Martin Vingron. "SVIM: structural variant identification using mapped long reads". In: *Bioinformatics* 35.17 (2019). eprint: https://academic.oup.com/bioinformatics/article-pdf/35/17/2907/29591954/btz041.pdf, pp. 2907–2915. ISSN: 1367-4803. DOI: 10.1093/bioinformatics/btz041. URL: https://doi.org/10.1093/bioinformatics/btz041.

[4] Tao Jiang et al. "Long-read-based human genomic structural variation detection with cuteSV". In: *Genome Biology* 21.1 (Aug. 2020), p. 189. ISSN: 1474-760X. DOI: 10.1186/s13059-020-02107-y. URL: https://doi.org/10.1186/s13059-020-02107-y.

[5] Szymon M. Kiełbasa et al. "Adaptive seeds tame genomic sequence comparison". In: *Genome Research* (2011). DOI: 10.1101/gr.113985.110. eprint: http://genome.cshlp.org/content/early/2011/02/04/gr.113985.110.full.pdf+html. URL: http://genome.cshlp.org/content/early/2011/02/04/gr.113985.110.abstract.

[6] Heng Li. "Minimap2: pairwise alignment for nucleotide sequences". In: *Bioinformatics* 34.18 (2018). eprint: https://academic.oup.com/bioinformatics/article-pdf/34/18/3094/25731860/bty191suppldata.pdf, pp. 3094–3100. ISSN: 1367-4803. DOI: 10.1093/bioinformatics/bty191. URL: https://doi.org/10.1093/bioinformatics/bty191.

[7] Jonathan LoTempio, Emmanuèle Délot, and Eric Vilain. "Benchmarking long-read genome sequence alignment tools for human genomics applications". In: *bioRxiv* (2021). Publisher: Cold Spring Harbor Laboratory _eprint: https://www.biorxiv.org/content/early/2021/07/11/2021.07.09.451840.full.pdf. DOI: 10.1101/2021.07.09.451840. URL: https://www.biorxiv.org/content/early/2021/07/11/2021.07.09.451840.

[8] Jingwen Ren and Mark J. P. Chaisson. "lra: A long read aligner for sequences and contigs". In: *PLOS Computational Biology* 17.6 (June 2021), pp. 1–23. DOI: 10.1371/journal.pcbi.1009078. URL: https://doi.org/10.1371/journal.pcbi.1009078.

[9] Fritz J. Sedlazeck et al. "Accurate detection of complex structural variations using single-molecule sequencing". In: *Nature Methods* 15.6 (June 2018), pp. 461–468. ISSN: 1548-7105. DOI: 10.1038/s41592-018-0001-7. URL: https://doi.org/10.1038/s41592-018-0001-7.

[10] Haojing Shao et al. "npInv: accurate detection and genotyping of inversions using long read sub-alignment". In: *BMC Bioinformatics* 19.1 (July 2018), p. 261. ISSN: 1471-2105. DOI: 10.1186/s12859-018-2252-9. URL: https://doi.org/10.1186/s12859-018-2252-9.

[11] Ivan Sović et al. "Fast and sensitive mapping of nanopore sequencing reads with GraphMap". In: *Nature Communications* 7.1 (Apr. 2016), p. 11307. ISSN: 2041-1723. DOI: 10.1038/ncomms11307. URL: https://doi.org/10.1038/ncomms11307.
